# Au_20_Ag_32_ Nanocluster Emitting Bright Near-Infrared-II Photoluminescence with Quantum Yield of 30% in Aerated Solution

**DOI:** 10.1021/acsnano.5c22362

**Published:** 2026-02-28

**Authors:** Avirup Sardar, Yitong Wang, Guiying He, Christopher G. Gianopoulos, D. Sulalith N. D. Samarasinghe, Zhongyu Liu, Kristin Kirschbaum, Christine M. Aikens, Rongchao Jin

**Affiliations:** 1 Department of Chemistry, 6612 Carnegie Mellon University, Pittsburgh, Pennsylvania 15213, United States; 2 Department of Chemistry and Biochemistry, 7923 University of Toledo, Toledo, Ohio 43606, United States; 3 Department of Chemistry, 5308 Kansas State University, Manhattan, Kansas 66506, United States

**Keywords:** atomically precise nanoclusters, Au−Ag alloy, near-infrared-II photoluminescence, thermally activated delayed fluorescence, phosphorescence

## Abstract

Atomically precise metal nanoclusters (NCs) have emerged as an important class of materials for optoelectronic applications, owing to their near-infrared-II (NIR-II) photoluminescence (PL) properties. To fully realize their applications, the PL quantum yield (PLQY) of NCs must be enhanced. In this regard, structure–property correlation studies are of critical importance. Herein, we report an alkynide-protected Au_20_Ag_32_ NC (charge neutral) protected by 36 ligands, including 12 Cl^–^ and 24 *p*-*tert*-butylphenylacetylide (^*t*^BuPA^–^). Structural analysis shows that the NC is a three-dimensional growth of a bi-icosahedral core. Theoretical analysis reproduces the experimental optical absorption spectral features. Interestingly, Au_20_Ag_32_ shows bright PL emission centered at 980 nm, with a PLQY of 30% in aerated and 33% in deaerated medium at room temperature, which is the highest among the reported NIR-II NCs. Furthermore, cryogenic PL measurements and transient absorption spectroscopy analysis reveal the PL mechanism, which involves both thermally activated delayed fluorescence (TADF) and phosphorescence (PH). This study is expected to motivate further research in expanding the Au–Ag nanoclusters and studying their high NIR-II emission.

## Graphical abstract

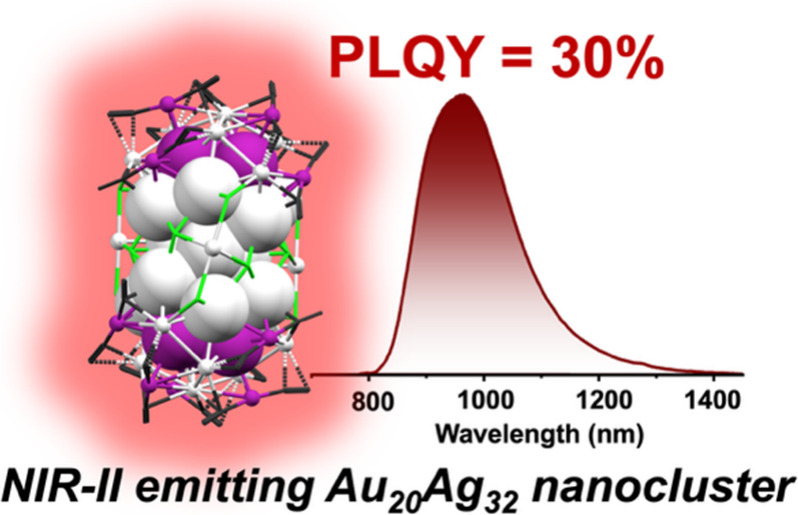

## Introduction

Near-infrared-II (NIR-II) emitting materials have recently been a captivating research topic due to the importance in bioimaging, phototherapy, solar energy conversion, optoelectronics, and fiber optics.^1–4^ In recent years, atomically precise metal nanoclusters (NCs) have emerged as a new class of NIR materials due to their tunable optical properties by structural control.^5–8^ While there has been significant research in designing NCs with tailored optical properties, NCs with a NIR-II response remain quite challenging. Thus, new synthetic strategies or postsynthesis engineering should be developed for achieving highly emissive NIR-II NCs.

Thiolate-protected metal NCs are being studied intensely, which has led to a quite clear picture about their structural evolution and how to tune their optical properties.^5,9–13^ In contrast, fewer systematic studies on the photophysical properties of alkynide-protected metal NCs exist.^14–16^ Unlike thiolates that bind with metals (e.g., M = Au and Ag) via σ-bonds in linear RS-M-SR or semiring RS-M-SR-M-SR motifs, alkynides can bond with metals via both σ and π-bonds due to the π electrons in the triple bond, leading to a diversity of bonding modes and opportunities for tailoring the photophysical properties.^17–21^ However, except for rare cases, the PLQY is generally not high enough for applications, especially in the NIR-II region. The NIR-II emissive NCs are of great interest due to their higher skin- or skull-penetrating capability compared to visible and NIR-I NCs, which is particularly appealing in biomedical research.^22^ NIR-II NCs are also attractive for light-emitting diode, laser, and solar energy conversion (e.g., the upconversion of NIR-II solar energy into the visible or NIR-I region for utilization by silicon solar cells).^1,5^

From the early work of NIR-emitting alkynide-protected Au NCs^23^ to recent time, considerable research has been done on improving the PLQY. Among the strategies such as the growth of longer or larger structures and the introduction of π-conjugated alkynes, doping/alloying is a crucial strategy that has been utilized to obtain NIR emissive NCs.^24–28^ In the literature, alkynide-protected homometallic Au, Ag, and Cu NCs have been studied extensively for their NIR emission.^28–30^ Reports have shown that doping/alloying of Cu into Au NCs may result in bright emission,^31,32^ and some of the NCs have also pushed from the NIR-I to the NIR-II region. Wang and Zheng groups have done seminal work on the synthesis and structure determination of Au–Ag alkynide NCs.^33–35^ Such NCs hold promise in tailoring their NIR optical properties for optoelectronic and catalytic applications.^14–16^

In this work, we report the discovery of an alkynide-protected Au_20_Ag_32_(^*t*^BuPA)_24_Cl_12_ NC (**Au**_**20**_ **Ag**_**32**_, ^*t*^BuPA: 4-*tert*-butylphenylacetylide). This M_52_ (M = Au and Ag) exhibits a structural growth around the bi-icosahedral M_23_ core in previous M_38_(SR)_24_ NCs but exhibits quite different optical absorption features. Theoretical analysis simulates its electronic structure and absorption spectrum. Significantly, Au_20_Ag_32_ shows strong NIR-II emission with a PLQY of 30% in ambient conditions and 33% in degassed solutions at room temperature, which is the highest PLQY among the reported NIR-II NCs. Detailed studies on the excited-state dynamics and temperature-dependent spectroscopic measurements reveal an intricate emission mechanism, which is a convoluted form of strong phosphorescence (PH) and weak thermally activated delayed fluorescence (TADF). This work demonstrates the promise of new designs for attaining highly NIR-II emissive NCs.

## Results and Discussion

### Synthesis, Purification, and Crystallization

The synthesis of the Au_20_Ag_32_ NC was conducted by modifying a previous one-pot procedure for alkynide-protected NCs.^36,37^ The procedure (Scheme 1 and Supporting Information) involves three primary steps: (1) reactions of Au­(I) and Ag­(I) salts with alkyne to form M­(I)–C≡CR complexes, where M = Au/Ag, (2) reduction of M­(I)–C≡CR complexes (where, R = ^*t*^BuPh– in this work) into NCs using a weak reducing agent such as *tert*-butylamine borane, and (3) growth into larger NCs by size-focusing/ripening under ambient conditions. Briefly, an equimolar amount of chloro­(dimethylsulfide)­gold­(I) (Me_2_SAuCl) and 4-*tert-*butylphenylacetylene (^*t*^BuPA) were dispersed in toluene, followed by the addition of triethylamine (NEt_3_). This led to the formation of a transparent yellow Au­(I)–C≡CR complex within a few minutes. After that, tetrakis­(acetonitrile)­silver­(I) tetrafluoroborate (Ag­(CH_3_CN)_4_·BF_4_) salt was added to the solution and sonicated for 10 min; note that this Ag­(I) salt is typically in large chunks and sparingly soluble in toluene, thus sonication is important to the formation of reddish yellow Au–Ag–C≡CR complexes. Then, the complexes were reduced using *tert*-butylamine borane ^*t*^BuNH_2_·BH_3_. The reaction was stopped after 4–5 h, and the reaction mixture was kept in the dark for 1 week, over which the NC growth/ripening occurred. It is important not to wash the reaction mixture because the unreacted precursors still play an important role in the formation of the desired product, Au_20_Ag_32_. After that, thin-layer chromatography (TLC) separation was performed, and the topmost dark band containing the target NC was collected (Figure S1). Rod-shaped dark black crystals of Au_20_Ag_32_ were obtained via a slow diffusion of methanol into a toluene solution of the NC at 4 °C within 2 weeks (Figure S2), followed by X-ray crystallography analysis.

**Scheme 1:**
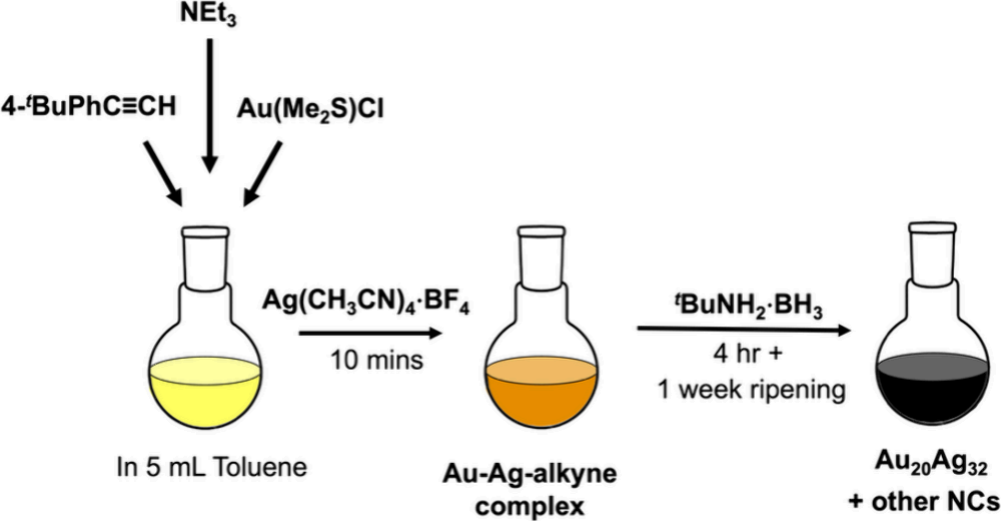
Synthetic Route for the Au_20_Ag_32_ NC

In most of the reported synthesis procedures for alkynide-protected Au–Ag or Au–Cu NCs, steps 1 and 2 mentioned above are generally involved. If we stopped at step 2, we found that Au_24_Ag_20_ and other Au NCs were obtained. Also, reaction overnight led to the formation of larger plasmonic nanoparticles. Thus, in our work, we introduced the ripening step (step 3) to obtain new NCs. The amount and form of the Ag precursor used is also critical for the synthesis of the target NC. We observed that using other Ag­(I) salts like AgBF_4_, AgNO_3_, or CH_3_COOAg does not afford the Au_20_Ag_32_ NC. Also, increasing or decreasing the ratio of Au/Ag from the optimized process has a large effect on the final product formation (Table S1).

Alkynide-protected alloy NCs, especially charge neutral ones, are notorious for being difficult to detect via electrospray ionization mass spectrometry (ESI-MS).^35,38^ In our case, positive-mode ESI-MS unfortunately shows no intact peaks; however, addition of cesium acetate (CsOAc) results in a prominent 3+ peak at *m*/*z* 4052.4 in agreement with the formula [Au_20_Ag_32_(C_12_H_13_)_24_Cl_13_Cs_4_]^3+^ and also a 2+ peak at *m*/*z* 6012.2 corresponding to [Au_20_Ag_32_(C_12_H_13_)_24_Cl_13_Cs_3_]^2+^ (Figure S3). These peaks are formed by the addition of four and three Cs^+^ ions from CsOAc and one Cl^–^ ion from the solvent to the native [Au_20_Ag_32_(C_12_H_13_)_24_Cl_12_]^0^ under ESI conditions, which is common in the case of alkynide-capped alloy NCs.^31^ The simulated isotope pattern of the formula matches well with the experiment (Figure S3 inset). Based on the ESI-MS and X-ray crystallography (note: multiple crystals give rise to the same unit cell), our product is pure and charge neutral, formulated as Au_20_Ag_32_(C_12_H_13_)_24_Cl_12_.

### Crystal Structure of Au_20_Ag_32_(^t^BuPA)_24_Cl_12_

The Au_20_Ag_32_ NC crystallizes in the monoclinic *C*2/*c* space group. A rod-shaped crystal with dimensions of 0.060 × 0.100 × 0.160 mm was used for the X-ray crystallographic analysis at 250 K (Tables S2–S4). The X-ray intensity data were measured (λ = 1.54178 Å) with a completeness of 99.3% (*R*_int_ = 8.21%, *R*_sig_ = 3.83%). The overall formula is determined to be Au_20_Ag_32_(C_12_H_13_)_24_Cl_12_. Structurally, the NC shows *C*_3_ symmetry and is composed of an elongated M_52_ metal core surrounded by 24 ^*t*^BuPA^–^ (gray wireframe) and 12 Cl^–^ (green) ligands (Figure 1a). The structure can be dissected in two ways: (1) the bi-icosahedral core and staple-motif view and (2) the tri-icosahedral top/bottom view.

**Figure 1:**
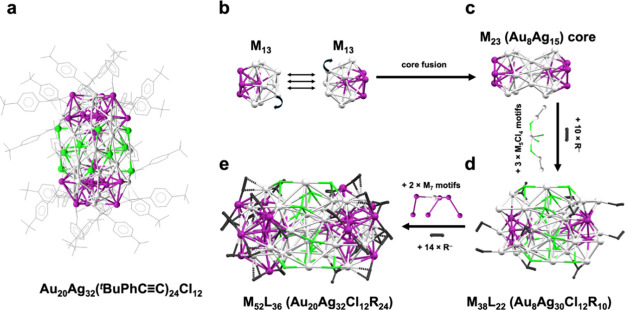
(a) Total structure of the Au_20_Ag_32_ NC, (b) face fusion of two distorted M_13_ icosahedral units, (c) bi-icosahedral M_23_ core, (d) addition of three M_5_Cl_4_ motifs to the belly of M_23_ (forming M_38_), and (e) addition of two M_7_ caps onto the left/right ends of M_38_ (forming M_52_). From panels (b) to (e), all carbons (except C≡C, black) are omitted for clarity (R = ^*t*^BuPhC≡C), color code: purple = Au, gray = Ag, green = Cl.

In the first view, we can describe the NC having a distorted, face-fused bi-icosahedral M_23_ (Au_8_Ag_15_) core (Figure 1b,c), which is capped by an M_15_ shell at the belly position and two M_7_ caps on the two ends of the M_23_ rod, forming the overall M_52_ structure. Although a few M_52_ NCs have been reported,^39–44^ they were mostly homometallic and of different structures. The M_23_ core is formed by the fusion of two M_13_ icosahedra, which is identical to Au_23_ and Ag_23_ cores (Figure S4) in the previous M_38_(SR)_24_ NCs;^45–47^ however, in our M_52_ NC, there is one more layer around the M_23_, thus it shows a rare case of three-dimensional structural “growth” around the stable M_23_ architecture.^48^ If the alkynides are taken into consideration, the M_23_ core is capped by an M_15_L_22_ [that is, Ag_15_Cl_12_(C≡CR)_10_] staple shell, making an overall M_38_L_22_ structure (Figure 1d). The difference between our NC and the classical M_38_(SR)_24_ NCs^45–47^ is two less ligands. This has important implications, as less ligands lead to the presence of 16 free electrons for the Au_20_Ag_32_ NC (i.e., 52­(Au 6*s* ^1^)–24­(alkynide)–12­(Cl^–^) = 16 e^–^), rather than 14 e^–^ as in the case of other M_38_ NCs with a bi-icosahedral core.^49^ As shown in Figure S17, the free electrons in the two sides of the NC do not interact as strongly as in the previous Au_38_(SR)_24_ or Ag_38_(SR)_24_,^45–47^ leading to less coupling between the superatomic P orbitals. In Figure S5, we compare the difference between alkynide-capped M_38_ vs thiolated Au_38_ NCs. Lastly, the M_38_ portion (Figure 1e) is further capped at its two ends by another two staple motifs each composed of M_7_L_7_ (Au_6_AgR_7_), forming an overall M_52_L_36_ structure.

The dimensions of the NC are 2.7 × 2.1 nm, which reduce to 1.38 × 0.86 nm when the ligand shell is removed (Figure S6a,b). The M_23_ core has an average Au–Au bond distance of 2.80 Å, Ag–Ag distance of 3.05 Å, and Au–Ag distance of 2.90 Å. (Figure S6c), which is comparable to the M_23_ in the literature.^45,50^ In general, the Au–Au and Au–Ag distances are comparable. In Au_20_Ag_32_, the belly M_15_ shell is made up completely of Ag atoms and has an average Ag–Ag distance of 3.11 Å (Figure S6d), and the two ends of M_7_ shells have significantly longer metal–metal distances of 3.57 and 3.11 Å for Au–Au and Au–Ag, respectively (Figure S6e).

The second view leads to cyclic tri-icosahedral top and bottom that are joined together by an Ag_6_Cl_12_ connector in the middle (Figure 2a,b). The top/bottom units are M_23_ (Au_10_Ag_13_), and each is formed by three layers: M_7_ (Au_1_Ag_6_), M_9_ (with an Au_3_ in the center, connected by three M_2_ (Au_1_Ag_1_)), and M_7_ (Au_3_Ag_4_) (Figure 2c). This M_23_ is comparable to the reported Au_23_ and M_23_ cores.^51–53^ Instead of being symmetric as in Au_23_, the M_23_ unit here is distorted due to the top/bottom asymmetric distribution of Au and Ag atoms, thus creating ring-strains. The Au–Ag bond lengths in this M_23_ are significantly longer than the Au–Au in Au_23_ and Au–Ag in M_34_ NCs, as shown in Figure S7. Nonetheless, as the two M_23_ are effectively mirror images of each other while being joined by the Ag_6_Cl_12_ connector (Figure 2b), the NC adopts an overall *C*_3_ symmetry.

**Figure 2:**
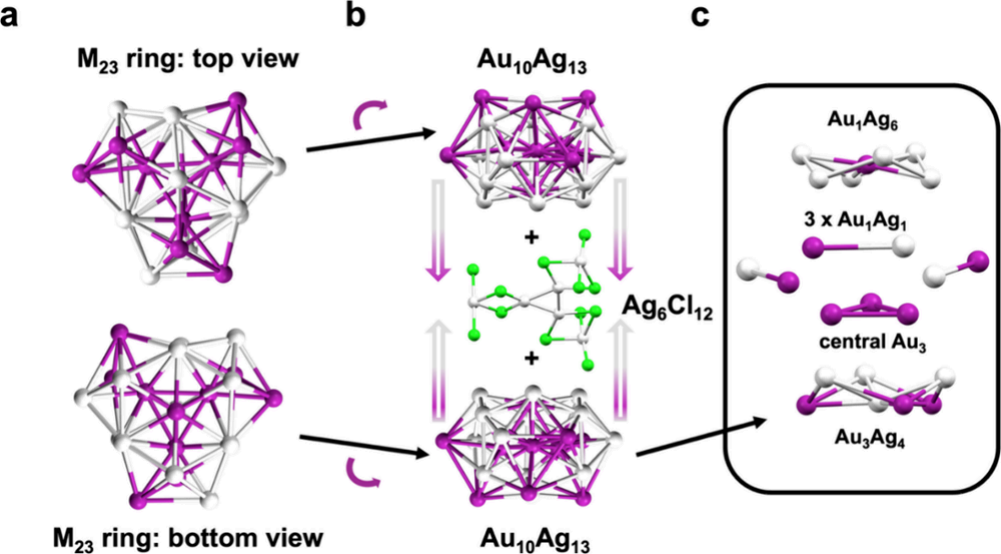
Structural anatomy of Au_20_Ag_32_ by the cyclic tri-icosahedral top/bottom view: (a) M_23_ ring, (b) joining of the two M_23_ rings with the Ag_6_Cl_12_ connector, (c) layer-by-layer dissection of the M_23_ ring. Color code: purple = Au, gray = Ag, green = Cl, all carbon atoms are omitted.

Focusing on the ligands, the M_52_ NC is protected by a total of 36 ligands (12 Cl^–^ and 24 ^*t*^BuPA^–^). Two types of chloride binding modes are found in the NC. Of these, six chlorides are arranged around the periphery in μ_3_ mode, whereas the other six chlorides are arranged in closer proximity to the core in μ_5_ binding mode (Figure S8). The chloride plays an important role in stabilizing the alkynide-protected alloy NCs, and the source of them in our work is the Au­(I) salt, as no chlorine-containing solvents were used in the synthesis. As discussed in the introduction, alkynes adopt binding modes in the NCs more diverse than those of thiols and phosphines. In our case, two types of alkynide attachments are present: (1) μ_3_-η^1^, η^1^, η^1^ mode and (2) μ_3_-η^1^, η^2^ mode (Figure S9). From the metal point of view, Ag atoms are as expected arranged with three types of linkages (RC≡C–Ag, (RC≡C)_2_–Ag, and (RC≡C)_3_–Ag)), whereas Au atoms only adopt a single (RC≡C–Au–C≡CR) linear linkage (Figure S10).

The well-ordered ligand arrangement and the stable M_23_ units (both views), along with a multitude of intracluster and intercluster interactions in the crystal packing shown in Figure S11, lead to the high stability of Au_20_Ag_32_ both in the crystal and solution phases. For example, after 1 month of storage under ambient conditions, no obvious signs of degradation of the NC were found, as the optical spectrum (Figure S12) remains the same.

### Optical Absorption and NIR-II Emission of Au_20_Ag_32_

The optical properties of the Au_20_Ag_32_ NC were investigated in dilute toluene solutions at room temperature and under ambient conditions unless otherwise noted. The absorption spectrum shows multiple peaks in the UV–vis-NIR range, including a peak at 365 nm, two convoluted peaks at 470 and 505 nm, followed by a broad peak at ∼600 nm and the longest wavelength peak at 835 nm (Figure 3a); the latter peaks generally correspond to electronic transitions between the NC core orbitals.^54^ The onset of absorption is ∼885 nm; thus, the optical gap (*E*_g_) is 1.4 eV (inset, Figure 3a). The absorption coefficient at 835 nm is 1.18 × 10^3^ M^–1^ cm^–1^ (Figure S13). Although the arrangement of the metal atoms in the NC resembles the core of Au_38_(SR)_24_ as discussed above, their spectra are different and peaks are much more pronounced in M_52_. The absorption peak positions of Au_20_Ag_32_ remain almost identical in different solvents (dichloromethane (DCM), chloroform, 2-methyltetrahydrofuran (2Me-THF), acetone, and hexane), indicating no charge transfer involvement in the electronic transitions.

**Figure 3:**
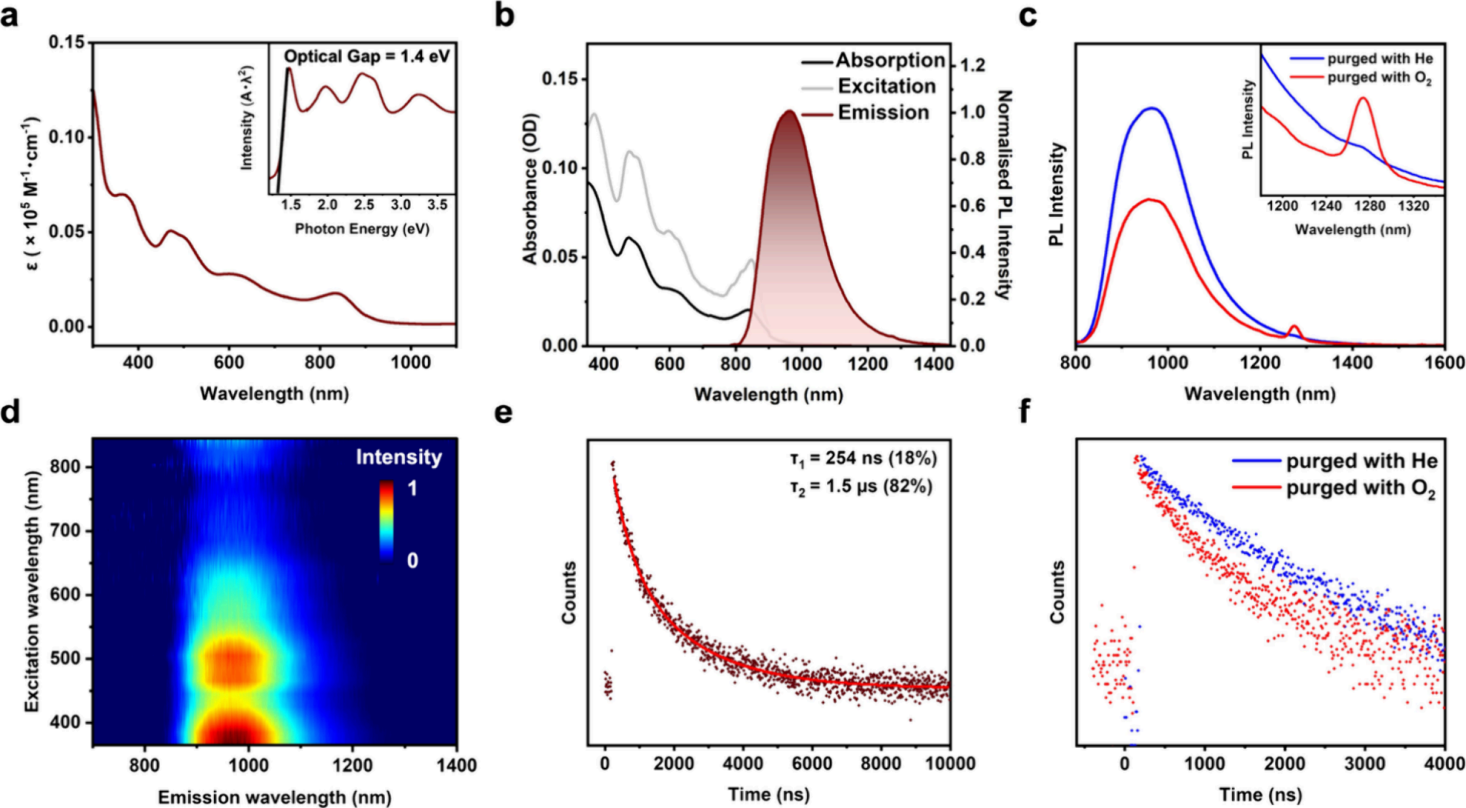
(a) UV–vis-NIR absorption spectrum of Au_20_Ag_32_; inset: absorption spectrum on the photon energy scale. (b) UV–vis-NIR absorption (black line), PL excitation (gray line), and PL emission (shaded) spectra of Au_20_Ag_32_. (c) PL spectra under He atmosphere (blue line) and O_2_ atmosphere (red line); inset: expanded regions showing the ^1^O_2_ phosphorescence emission. (d) Excitation–emission contour map of Au_20_Ag_32_. (e) Emission lifetime decay profile. (f) Emission lifetime decay profile under He atmosphere (blue) and O_2_ atmosphere (red). All spectra were measured using CDCl_3_ solutions of the NC to avoid solvent absorption in the NIR.

Interestingly, we found that Au_20_Ag_32_ gives strong NIR-II emission (peak at 980 nm) upon excitation at 375 nm with a PLQY of 30%. When measured in deaerated deuterated chloroform (CDCl_3_) solvent (purged with He/N_2_), the PLQY increases to 33% (Figure 3b and Figure S14). To the best of our knowledge, this is the highest quantum yield of reported NCs in the NIR-II region (Table S5). The PLQY values of Au_20_Ag_32_ are determined by an absolute method using an integrating sphere and further verified using Au_42_ NC as a reference (Figures S15 and S16a). The PL excitation (PLE) tracks the absorption profile (Figure 3b), indicating that the emission is from the NC, rather than any impurities. The excitation–emission map shows that NC has similar emission profiles when excited across a wide range of wavelengths (Figure 3d). Furthermore, if we look at the PL spectrum carefully, we observe a small shoulder at 900 nm apart from the primary 980 nm peak, and this shoulder becomes more pronounced when measured in a nonpolar solvent (e.g., hexane) but disappears when measured in a highly polar solvent (e.g., acetone) (Figure S16b,c).

The PL lifetime shows a biexponential decay, with the two components being 254 ns (photon counts: 18%) and 1.5 μs (82%) (Figure 3e). These features indicate that the NC possesses a complicated emission mechanism (see sections below). To ascertain the nature of the emission, we measured the PL spectra in He-purged and O_2_-purged CDCl_3_ solvents. For the O_2_-saturated solution, we observed that the PL intensity decreases with the appearance of a small distinct peak around 1275 nm, which is attributed to the generation of singlet oxygen with its phosphorescence at 1275 nm (Figure 3c). This, along with the decrease of the average lifetime (1.85 μs to 990 ns) suggests a triplet-singlet energy transfer (TSET) process between Au_20_Ag_32_ and ^3^O_2_ (Figure 3c,f). Referring to previous works,^23,25,55^ along with the two lifetimes and their values under ambient conditions (lifetime >1 μs is generally attributed to phosphorescence in the NCs), it is safe to assign phosphorescence (PH) and TADF as the emission pathways in Au_20_Ag_32_. The presence of a PL hump in nonpolar solvents also indicates the presence of two emission bands, i.e., two emission states.

### Theoretical Analysis of Electronic Structure and Optical Absorption

This M_52_ nanocluster exhibits distinctive electronic structure characteristics in the HOMO–LUMO region, with a calculated HOMO–LUMO gap of 1.15 eV (Figure 4a,b). Analysis of the molecular orbitals reveals that both HOMO and LUMO electron densities are mainly located around the metal core of the NC (Figure 4c), displaying nodal structures, with the HOMO exhibiting three main nodes (four lobes; with σ* symmetry between two superatomic P orbitals), while the LUMO displays four nodes (five lobes; with σ symmetry between two superatomic D orbitals). To obtain a detailed description of the electronic structure of the NC, molecular orbital analysis (Figure S17 and Table S6) and Hirshfeld charge analysis (Figure S18) are presented in the Supporting Information. To gain a theoretical understanding of the absorption spectrum of the Au_20_Ag_32_ nanocluster, time-dependent density functional theory plus tight binding (TDDFT+TB) calculations were performed. Figure 4a illustrates the theoretical absorption spectrum (red) in comparison to that of the experiment (cyan). All the theoretical peaks are red-shifted, which is expected for the GGA functionals used in the computations.^56^ Analysis of these excitations reveals that each peak involves multiple electronic transitions; thus, Figure 4b and Table S7 provide a detailed analysis of the prominent peaks and their electronic transition character.

**Figure 4:**
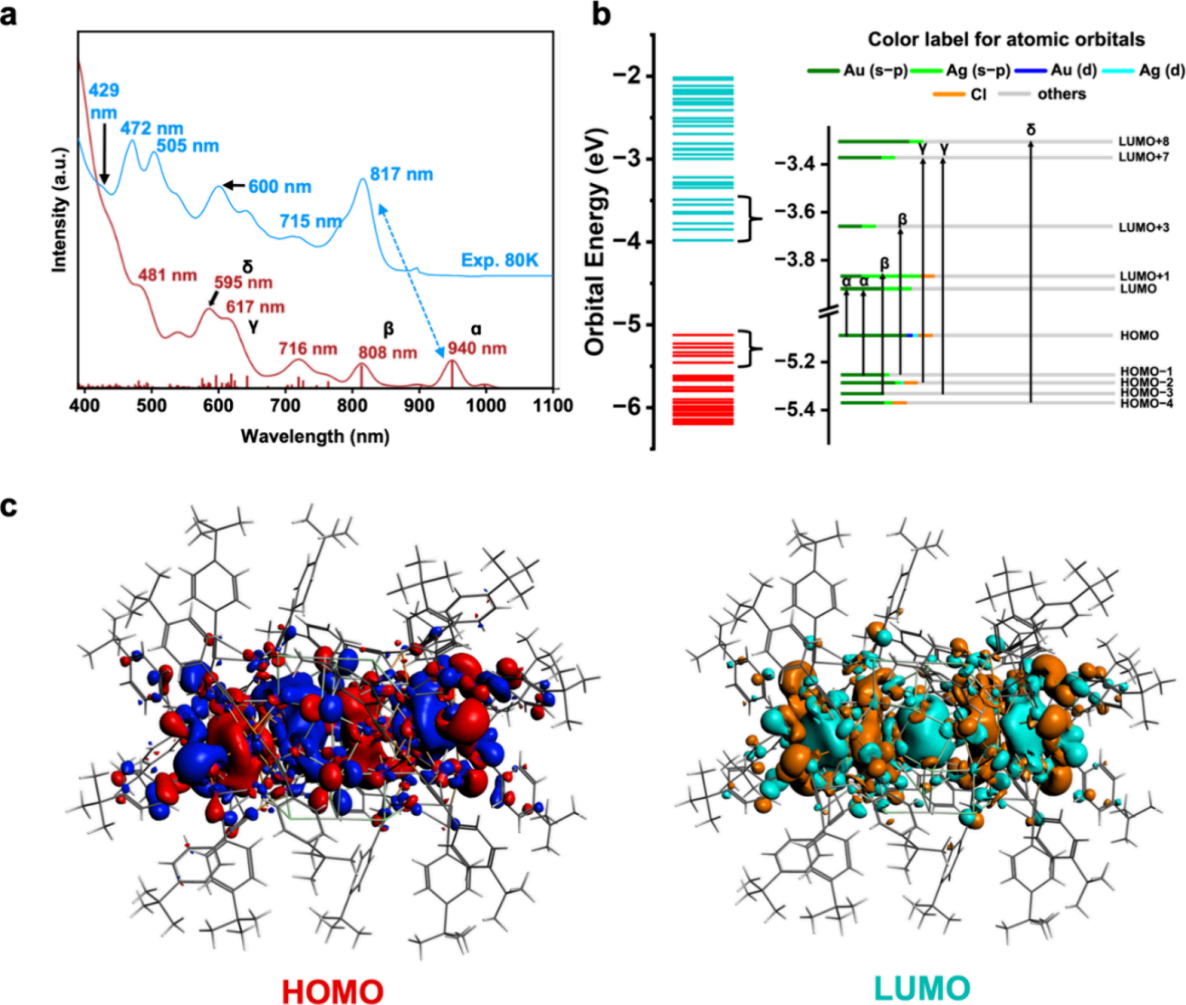
(a) Simulated optical absorption (red) spectrum of Au_20_Ag_32_ with annotated peaks of highest oscillator strengths and the experimental fine structure absorption spectrum at 80 K (cyan). (b) Energy diagram of Kohn–Sham molecular orbitals and associated components of important atomic orbitals. (c) Distributions of the frontier molecular orbitals of Au_20_Ag_32_.

Although red-shifted, the simulated absorption spectrum closely resembles the experimental one and exhibits characteristic peaks in the 400–1000 nm region. Among these bands, the main peak of interest is calculated to be at 940 nm (1.32 eV), which underestimates the cryogenic experimental peak at 817 nm (1.52 eV) by 0.198 eV (Figure 4a). The excitation involves a dominant transition from HOMO to LUMO (52.0%) and HOMO–1 to LUMO (25.3%), which are labeled as α in Figure 4a,b. These electronic transitions exhibit metal-centered characteristics. Therefore, peak α is a result of metal-center→metal-center electronic transitions. The experimental peaks δ at 472 nm (2.63 eV) and γ at 505 nm (2.45 eV) correspond to the calculated transitions at 595 nm (2.08 eV) and 617 nm (2.01 eV). These peaks are underestimated by ∼0.4 eV, which is still reasonable for GGA functionals. Molecular orbital analysis (Figure 4b) reveals that these transitions are also of metal-center to metal-center character. Overall, the simulated spectrum is very similar to the fine structure spectrum of the cryogenic absorption.

### Cryogenic Emission and Absorption Studies for Understanding the PL Mechanism

To understand the emission mechanism of Au_20_Ag_32_ and details about its radiative/nonradiative pathways, temperature-dependent steady-state PL measurements were carried out with a cryostat from 298 K (room-temperature) to 80 K (liquid N_2_) in 2-MeTHF (clear glass formation at low temperatures). From Figure 5a, we can see that with excitation at 375 nm, the PL peak becomes sharper and the PLQY increases to 78% at 80 K (three times the room-temperature PLQY 26% in 2-MeTHF). The sharpening and enhancement of PL are a direct result of the reduction of metal–metal and metal–ligand bond vibrations at low temperatures. Generally, the increase of PL intensity is attributed to the reduction of nonradiative relaxation and/or the increase in the radiative relaxation pathways.^57^ The average lifetime prolonging from 1.27 μs at 298 K to 8.3 μs at 80 K (Figure 5d) indicates that the excited state becomes long-lived due to the reduced vibrations. The UV–vis-NIR spectrum of Au_20_Ag_32_ remains unchanged before and after the cryogenic experiments (Figure S19), indicating no photoinduced degradation.

**Figure 5:**
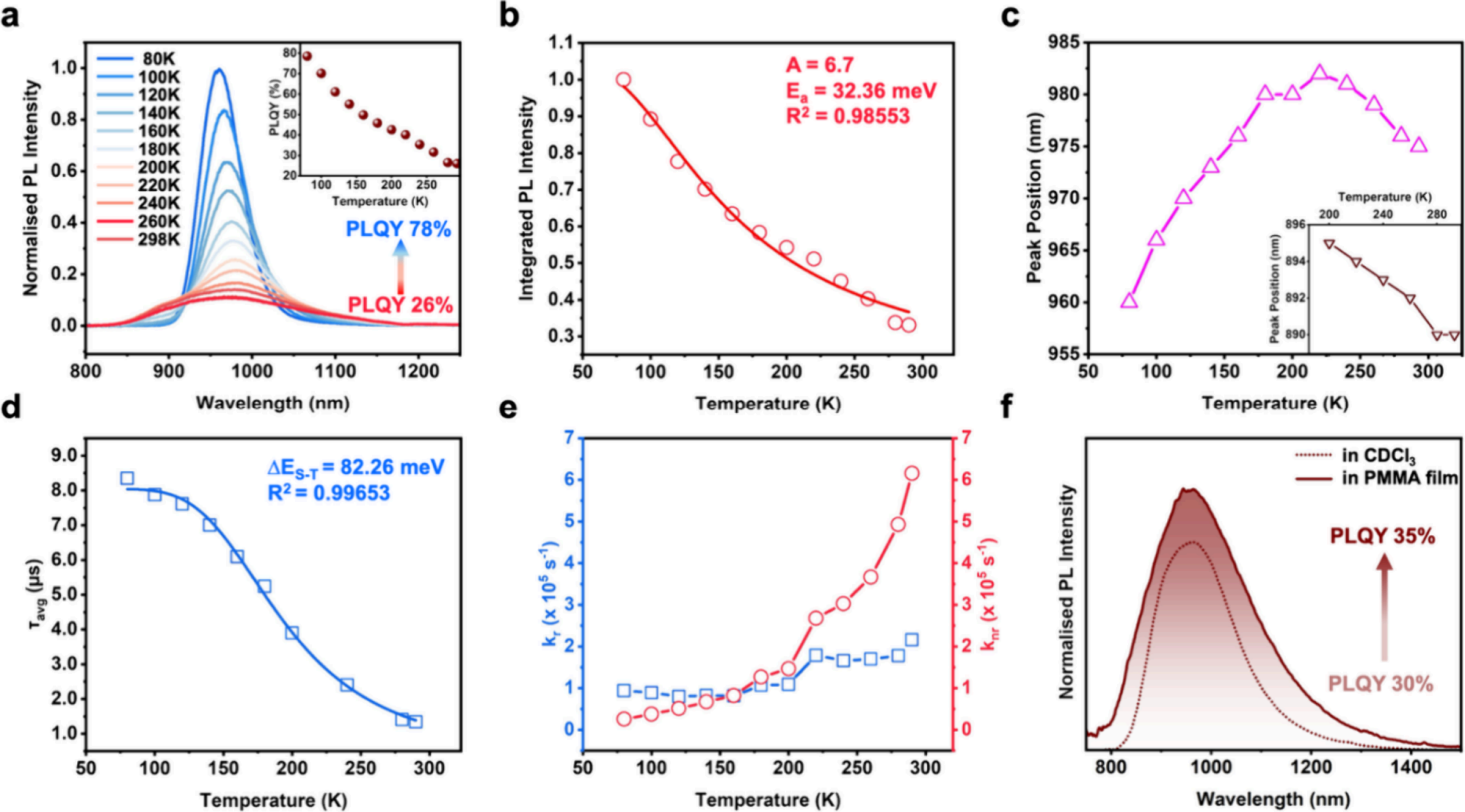
(a) Temperature-dependent PL spectra of Au_20_Ag_32_ in 2-methyltetrahydrofuran; inset: change in PLQY as the temperature is decreased from 298 to 80 K. (b) Normalized integrated PL intensities at different temperatures (the line shows the fitting using eq 1). (c) Peak position of the main PL peak at different temperatures; inset: peak position of the shoulder band at different temperatures (note: the shoulder vanishes <180 K). (d) Temperature-dependent emission lifetimes of Au_20_Ag_32_ (the line shows the fitting using eq 2). (e) Plot of radiative decay rate constants (blue) and nonradiative decay rate constants (red) from 298 to 80 K. (f) PL spectra of the NCs embedded in PMMA film (shaded) and in solution state (dotted line).

To investigate the radiative and the nonradiative pathways, we plotted the temperature-dependent PL intensities (with the PL intensity at 80 K normalized as unity) (Figure 5b). Previous studies^58–60^ have shown that details about the nonradiative and radiative pathways can be extracted using an Arrhenius-type equation:^61^

$$I\left(T\right)=\frac{I\left(0\right)}{1+Ae^{-E_{a}/k_{B}T}}$$ 1

where *I*(0) is the PL intensity at the lowest theoretically possible temperature (0 K), *A* is the ratio of nonradiative to radiative probability, and *E*_a_ is the activation energy of the quenching pathway (nonradiative channel in our case). It is important to note that, for our data, we assume only one nonradiative relaxation pathway as the dominant quenching channel. By fitting the data with eq 1, we find the *E*_a_ to be 32.24 meV (i.e., phonon energy of 260 cm^–1^) and the nonradiative/radiative probability ratio *A* to be 0.98.

In addition, Figure 5c shows that the peak first redshifts from 975 to 980 nm with temperature drop and then gradually blue-shifts to 960 nm down to 80 K (the shoulder at 896 nm also redshifts to 900 nm before disappearing as shown in the inset of Figure 5c and Figure S20); note that the PLE spectra show no such shift (Figure S21) and all of them track the absorption profile. This zigzag pattern of PL peak shift is indicative of TADF.^57,62,63^

Most interestingly, we observed that when the temperature was lowered from 298 to 160 K, the shoulder band at ∼900 nm (Figure 5a) along with the main PL peak of Au_20_Ag_32_ remains prominent but disappears at 140 K and below, and the overall peak becomes sharp and single. We performed peak deconvolution using the pseudo-Voigt function (Figure S22) and found that the peak contribution from the shoulder decreases and disappears at 160 K, whereas the contribution of the main peak increases and becomes the only one at temperatures <160 K. We attribute the shoulder (or the deconvoluted smaller peak) as the TADF component, which is sensitive to the thermal energy around the system. In the case of pure phosphorescence (PH), after initial photoexcitation, the electron relaxes from higher excited states (S_*n*_) to the lowest singlet excited state (S_1_) and then it generally undergoes intersystem crossing (ISC) to the triplet T_1_ state and emits from there. In various luminophores, especially in the NCs, at higher temperatures, the electron acquires enough thermal energy to undergo reverse intersystem crossing (RISC) from T_1_ back to the S_1_ state, thereby exhibiting TADF together with PH, which is the Au_20_Ag_32_ case. However, at lower temperature (<160 K) for Au_20_Ag_32_, electrons can undergo intersystem crossing (downhill) to the T_1_ state, but the uphill RISC pathway becomes suppressed; thus, the TADF disappears, and one observes PH only. This is further corroborated by the emission lifetime values (Figure S23), where one can see that the PL decay curves are biexponential at higher temperatures but become monoexponential at lower temperatures.

From the average PL lifetimes and the PLQY values, we can calculate the radiative rate (*k*_r_) and nonradiative decay rates (*k*_nr_) (Table 1 and Figure 5e). We observe that, as the temperature is decreased from 298 to 80 K, *k*_r_ decreases to 44% of the value at 298 K, whereas *k*_nr_ decreases drastically to around 4% of its initial value at 298 K. These observations agree with the observation that the quenching of nonradiative pathway at low temperatures leads to sharper peaks and higher PLQY. Also, if we plot the full width at half-maximum (fwhm) of the emission peak at varying temperatures (Figure S24), we obtain a linear relationship between fwhm vs *T*, which indicates weak electron–phonon coupling^55^ and hence strong PL, and the plateaus toward the highest and the lowest temperature regions, being another identifying feature of TADF.^64^

**Table 1:** PLQY (Φ_PL_), Average Lifetime (τ_avg_), Radiative (*k*_r_), and Non-Radiative Decay (*k*_nr_) Rate Constants Extracted from the Cryogenic Data for Au_20_Ag_32_ Solution in 2-MeTHF

temperature	Φ_PL_ (%)	**τ**_**avg**_ **(ns)**	**τ**_**1**_ **(ns)**	**τ**_**2**_ **(ns)**	*k*_r_ (s^–1^)^a^	*k*_nr_ (s^–1^)^b^
80 K	78.5	8347	8347 (100%)		9.41 × 10^4^	2.57 × 10^4^
100 K	70.1	7874	7874 (100%)		8.90 × 10^4^	3.80 × 10^4^
120 K	61.0	7609	7609 (100%)		8.02 × 10^4^	5.13 × 10^4^
140 K	55.0	6677	6677 (100%)		8.25 × 10^4^	6.73 × 10^4^
160 K	49.8	6091	6091 (100%)		8.17 × 10^4^	8.25 × 10^4^
180 K	45.8	4283	4320 (99%)	552 (1%)	1.07 × 10^5^	1.27 × 10^5^
220 K	40.1	2235	2324 (95%)	534 (5%)	1.79 × 10^5^	2.68 × 10^5^
260 K	31.6	1823	1954 (92%)	312 (8%)	1.70 × 10^5^	3.67 × 10^5^
298 K	26	1202	1355 (86%)	262 (14%)	2.16 × 10^5^	6.16 × 10^5^

a *k*_r_ = Φ_PL_ **·**τ_avg_^–1^.

b Calculated by *k*_nr_ = (1 – Φ_PL_)**·**τ_avg_^–1^.

In addition to the cryogenic measurements, we also measured the PL and lifetime of the NCs embedded in the poly­(methyl methacrylate) (PMMA) matrix. Previously, it was shown that polymer encapsulation generally increases the PL intensity of NCs due to the suppression of *k*_nr_ or the nonradiative decay pathways.^65,66^ In the current work, we also found that the PLQY of Au_20_Ag_32_ increases to 35% (c.f., 30% in CDCl_3_ solution) (Figure 5f). The average lifetime of the NCs in PMMA shows a slight increase to 1.38 μs (Figure S25), but interestingly, the contribution of the faster component (323 ns) decreases to ∼8% and the slower component (1.4 μs) increases to ∼92% from solution-state values of 18 and 82%, respectively. This is consistent with the PL mechanism having two emissive states (singlet and triplet) with a small energy gap. In the film state, the S_1_ and T_1_ energy levels of Au_20_Ag_32_ become much closer to each other, leading to more efficient ISC and therefore higher PLQY as well as greater contribution of PL from the T_1_ state.

We further estimate the singlet–triplet energy gap (the S_1_–T_1_ gap) by plotting the average lifetime of the emission vs *T* for fitting with a Boltzmann-type equation.^67^ Just like complexes, NCs show quantized energy levels, and thus assumptions considered for the complexes are also applicable to NC systems.^66^ If we consider S_1_ and T_1_ as the only emissive states, along with fast thermalization between them, the Boltzmann-type equation can be expressed as

$$\tau_{avg}=\frac{1+e^{-\Delta E_{S-T}/k_{B}T}}{\frac{1}{\tau \left(T_{1}\right)}+\frac{1}{\tau \left(S_{1}\right)}e^{-\Delta E_{S-T}/k_{B}T}}$$ 2

where Δ*E*_S–T_ is the energy gap between the T_1_ and S_1_ states, τ­(T_1_) and τ­(S_1_) are their intrinsic decay times, and *k*_B_ is the Boltzmann constant. If we plot the average lifetime values against *T*, we observe a sigmoidal curve, and data fitting with eq 2 gives Δ*E*_S–T_ of 82.3 meV for Au_20_Ag_32_. Typically, NCs with small Δ*E*_S–T_ (e.g., Au_22_ = 37 meV, Ag_22_ = 107 meV, Au_52_ = 111 meV) can undergo rapid RISC and therefore exhibit TADF, as is the current case. For NCs with Δ*E*_S–T_ > 150 meV, such as the Au_42_ quantum rod,^59^ the RISC barely occurs, leading to nearly exclusive prompt fluorescence (PF) and/or PH.

We also performed temperature-dependent absorption spectral measurements of Au_20_Ag_32_ (dissolved in 2-MeTHF) from 293 down to 80 K, primarily to normalize the quantum yield values at each temperature since the absorption is somewhat enhanced at low temperatures. One can see that the lowest energy peak exhibits a distinct blue shift (Figure S26) as well as an increase in intensity as the temperature decreases. Ramakrishna et al.^68,69^ and Liu et al.^66^ previously explained the temperature-dependent blue shift of the HOMO–LUMO peak for Au_25_ and Au_38_ in terms of electron–phonon coupling. Following their approach, we used a modified Bose–Einstein single oscillator model^68^ to fit the temperature dependence of the lowest-energy absorption peak of Au_20_Ag_32_, and the simplified single oscillator model can be written as

$$E\left(T\right)=E\left(0\right)-\left\langle C\right\rangle \left\langle \hbar \omega \right\rangle \left[\coth\left(\frac{\left\langle \hbar \omega \right\rangle }{2k_{B}T}\right)-1\right]$$ 3

where ⟨ℏω⟩ represents the *average* phonon energy of vibrational modes, ⟨*C*⟩ is the coupling constant which indicates the electron–phonon coupling strength, and *E* (T) is the corresponding electronic transition gap at *T*. To avoid inaccuracies and errors arising from reading out the *E*_g_ (i.e., at zero absorbance of the spectrum), we use the peak position, instead of the onset of absorption, for data analysis. Also, to have accurate peak position values, we convert the temperature-dependent absorption spectra to photon energy scale, i.e., the plot of absorption intensity (*A*·λ^2^, where *A* is the absorbance and λ is the wavelength) vs photon energy (eV). The lowest-energy peak blueshifts from 1.47 to 1.52 eV (Figure S27), and data fitting with eq 3 (Figure S28) gives rise to the average phonon energy, coupling constant, and the energy gap as 15.6 ± 2 meV, 1.322 ± 0.05 and 1.541 eV, respectively. Interestingly, although the coupling constant of Au_20_Ag_32_ is comparable to the reported values for Au_25_ and Au_38_,^68,69^ the average phonon energy is ∼3-fold lower. Previous works on Au_52_ have shown that the difference in the structure and ligands plays important roles in determining the phonon energy of the vibrational modes.^65,70^

### Transient Absorption Analyses

To further confirm our proposed mechanism, we probed the excited-state dynamics of Au_20_Ag_32_ using both nanosecond transient absorption (ns-TA) and femtosecond transient absorption (fs-TA) spectroscopies. Upon photoexcitation at 365 nm, three ground-state bleach (GSB) signals were observed around 475, 500–525, and 590–650 nm within the fs-TA wavelength window (Figure 6a and Figure S29). These are consistent with the steady-state absorption of the NC; note that ns-TA expands the window and thus observes the NIR GSB signal at 835 nm that matches the steady-state HOMO–LUMO transition. Additionally, two excited-state absorption (ESA) signals were observed around 525–600 nm and from 650 nm to the NIR. The high excited states decay in several picoseconds, and the following excited state extends from the picosecond to microsecond time scale.

**Figure 6:**
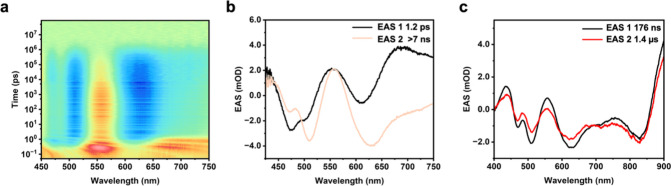
(a) Transient absorption data map of Au_20_Ag_32_ pumped at 365 nm (in toluene). (b, c) Global fitting analysis and evolution associated spectra for the fs-TA and ns-TA data, respectively, giving rise to species of 1.2 ps, 176 ns, and 1.4 μs.

Furthermore, we performed global fitting analysis of the TA spectra to extract the excited-state time constants. In the fs-TA, two evolution processes were found, with one component of 1.2 ps and the other being ≫7 ns (Figure 6b). The 1.2 ps component is attributed to the convolution of internal conversion and intersystem crossing. (Note: The reverse intersystem crossing should be consistent with the TADF time constant.) The spectra of the long-lived state observed in fs-TA are quite similar to the spectra from ns-TA. Also, in ns-TA, two species evolved with similar profiles (Figure 6c). The faster component (176 ns) is assigned to the reverse intersystem crossing, which results in the delayed fluorescence, and the slower component (1.4 μs) is attributed to the relaxation of the triplet state related to the phosphorescence. These values are consistent with the two components from emission lifetimes. Thus, the transient absorption measurements verify the proposed mechanism discussed in the above sections of PL properties.

Based on the steady-state PL results and the excited state dynamics, we summarize the emission mechanism of Au_20_Ag_32_ (Scheme 2). At higher temperatures (rt to 160 K), both TADF and PH are observed as RISC is feasible. At temperatures below 160 K, the uphill RISC pathway is blocked due to the lack of sufficient thermal energy, leading to PH only. The ISC occurs within 1.2 ps, but its accurate time cannot be resolved, whereas RISC occurs significantly slower, with its time constant being 176 ns.

**Scheme 2:**
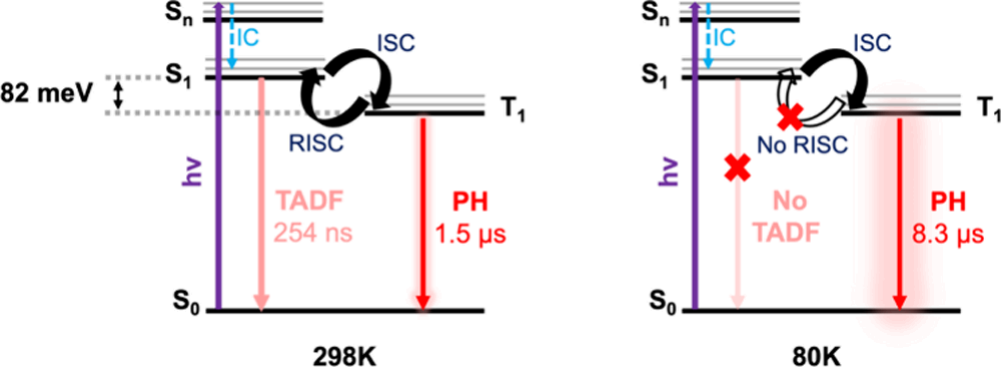
Proposed PL Mechanism of Au_20_Ag_32_ (IC = Internal Conversion, ISC = Intersystem Crossing, RISC = Reverse Intersystem Crossing)

## Conclusions

In summary, we have synthesized a new alkynide-protected Au_20_Ag_32_ (M_52_) NC by employing a modified synthetic approach, particularly with a ripening step favoring the NC growth. Single-crystal X-ray diffraction revealed the NC structure with an unprecedented M_52_ architecture. Based on the structure, theoretical simulations reproduce the experimental absorption fine structure. The NC shows bright PL emission in the NIR-II region with 30% PLQY in ambient conditions and 33% in degassed medium, and 35% when embedded in the PMMA matrix, which is the highest reported PLQY for NCs with emission peak wavelengths longer than 950 nm. Based on the cryogenic measurements as well as transient absorption spectroscopy, the PL is composed of thermally activated delayed fluorescence and phosphorescence. Overall, we hope this work not only acts as a major step toward the discovery of large bimetallic nanoclusters with new core architectures but also provides guidance toward future research on NIR-II emitting materials.

## Methods/Experimental Section

The synthesis of the Au_20_Ag_32_ nanocluster followed a reduction of Au­(I)–Ag­(I)-alkynide (4-*tert*-butylphenylacetylene) complexes by *tert*-butylamine borane. After the reaction (4–5 h), the mixture was ripened in the dark for 1 week. The target product was isolated by thin-layer chromatography. Crystallization was performed by slow diffusion of methanol into a toluene solution of the nanocluster at 4 °C within 2 weeks. Details are provided in the Supporting Information.

## Supplementary Material



## Notes

Details of the Au_20_Ag_32_ synthesis, characterization, X-ray crystallography, cryogenic emission studies and transient absorption analyses, molecular orbital characteristics, and Hirshfeld charge distribution (PDF)
