# Supplementary material for: Au20Ag32 Nanocluster Emitting Bright Near-Infrared-II Photoluminescence with Quantum Yield of 30% in Aerated Solution
Source: ACS Nano. 2026 Feb 28;20(10):8827–37. doi: 10.1021/acsnano.5c22362 (PMC13001081; doi:10.1021/acsnano.5c22362)
Supplement: Supplementary file 1 [file nn5c22362_si_001.pdf]

Supporting Information:

$Au_{20}Ag_{32}$ Nanocluster Emitting Bright NIR-II Photoluminescence with Quantum Yield of 30% in Aerated Solution

Avirup Sardar,[1] Yitong Wang,[1] Guiying He,[1] Christopher G. Gianopoulos,[2] D. Sulalith N. D. Samarasinghe,[3] Zhongyu Liu,[1] Kristin Kirschbaum,[2] Christine M. Aikens,[3] and Rongchao Jin[1]*

[1]Department of Chemistry, Carnegie Mellon University, Pittsburgh, Pennsylvania 15213, United States
[2]Department of Chemistry and Biochemistry, University of Toledo, Toledo, Ohio 43606, United States
[3]Department of Chemistry, Kansas State University, Manhattan, Kansas 66506, United States

*Corresponding author, email: rongchao@andrew.cmu.edu

S1. EXPERIMENTAL

S1.1. Chemicals

Tetrachloroauric (III) acid ($HAuCl_4 \cdot 3H_2O$, 99.999% metal basis, Sigma-Aldrich), dimethyl sulfide (SMe_2, >99%, Sigma-Aldrich), 4-*tert*-butylphenylacetylene ($^tBuPhC{\equiv}CH$, 96%, Thermo Scientific Chemicals), tetrakis(acetonitrile)silver(I) tetrafluoroborate ($Cu(CH_3CN)_4BF_4$, 98%, Sigma-Aldrich), triethylamine (NEt_3, for synthesis, Sigma-Aldrich), *tert*-butylamine borane ($(CH_3)_3CNH_2 \cdot BH_3$, powder, 97%, Sigma-Aldrich). Solvents: ethanol, ethyl ether, toluene, methanol, dichloromethane (DCM), *n*-hexane, chloroform, acetone, 2-methyl tetrahydrofuran, chloroform-d and toluene-d_8 (HPLC grade for all solvents). All chemicals were used as received without further purification. Thin-layer chromatography (TLC) plates were purchased from iChromatography (silica gel, 250 μm).

S1.2. Synthesis Procedures

Synthesis of chloro(dimethylsulfide)gold(I) (AuCl(SMe_2))

$HAuCl_4 \cdot 3H_2O$ (500 mg, 1.27 mmol) was dissolved in ethanol (20 mL), followed by the addition of SMe_2 (280 μL, 3.81 mmol), and the solution was vigorously stirred for 2 hours. After stirring, the white precipitate was collected by centrifugation, which was washed with ethyl ether and finally dried to give the product as a white powder

Synthesis of $Au_{20}Ag_{32}(C_{12}H_{13})_{24}Cl_{12}$

In a 25 mL round-bottom flask, 29.4 mg (0.1 mmol) chloro(dimethylsulfide)gold(I) [(AuCl(SMe_2))] was dispersed in 5 mL toluene. To that 18 μL (0.1 mmol) of 4-*tert*-butylphenylacetylene was added before the addition of 50 μL triethylamine (NEt_3). The reaction mixture was vigorously stirred for 10 min and the solution became transparent yellow in color. After that, 18 mg (0.05 mmol) tetrakis(acetonitrile)silver (I) tetrafluoroborate [($Ag(CH_3CN)_4 \cdot BF_4$] was added and the reaction mixture was sonicated for 10 min, during which the solution turned turbid orange. Next, 1.7 mg of borane *tert*-butylamine [$(CH_3)_3CNH_2 \cdot BH_3$] (dissolved in 1 mL ethanol) was added dropwise to the reaction mixture under vigorous stirring. Within a few seconds, the solution turned black, and the reaction was allowed to proceed in the dark. After 4-5 hours, the

reaction was stopped and kept in a cool, dry and dark place for one week. Then, the solvent was removed via rotary evaporation, and the crude product was washed with methanol, extracted using dichloromethane and concentrated for TLC separation. The mixture of NCs was pipetted onto a TLC plate, and the separation was conducted in a developing tank (developing solvent 1:3 (v/v) DCM:*n*-hexane). The topmost dark band was cut off and dissolved in DCM for initial characterization. Rod-shaped single crystals of $Au_{20}Ag_{32}$ were obtained via vapor diffusion of methanol or acetonitrile into a toluene solution of the NC at 4 °C for 2-3 weeks, followed by X-ray crystallography analysis. The crystals were dissolved in suitable solvents for further spectroscopic experiments. The yield was ~10% (Au atom basis)

S1.3. Characterization

Steady-State UV-Vis-NIR Measurements
The UV–Vis–NIR spectra of $Au_{20}Ag_{32}$ solutions were measured on a UV-3600 Plus spectrophotometer (Shimadzu) with a wavelength range of 185-3300 nm.

Electrospray Ionization Mass Spectrometry (ESI-MS)
ESI-MS were performed on a Waters Q-TOF mass spectrometer equipped with Z-spray source. The sample was dissolved in DCM and diluted (2:1 v) by dry methanol containing 50 mM cesium acetate (≥99.99%, Aldrich). The source temperature was kept at 70 °C. The sample was directly infused into the chamber at 5 μL/min. The spray voltage was kept at 2.20 kV and the cone voltage at 60 V.

Steady-state Photoluminescence Measurements
Steady-state photoluminescence spectra were measured using an Edinburgh FLS-1000 spectrofluorometer. Near-infrared PL was measured using a wide range InGaAs-based PMT-1700 detector (wavelength range: 500-1650 nm) cooled by liquid nitrogen down to -80℃. Cryogenic PL was done on a home-built low temperature system, including the FLS-1000 spectrofluorometer, a vacuum pump, an Optistat CF2 cryostat (Oxford Instruments) and a temperature controller. Liquid nitrogen was used as the cryogen.

Time-Resolved Photoluminescence Measurements
Time-correlated measurements were performed using an EPL-450 picosecond pulsed diode laser (Edinburgh Instruments) in multi-channel scaling (MCS) mode. The excitation wavelength from EPL-450 was 450 nm with a variance smaller than 5 nm. The pulse frequency was varied in the range of 100 kHz – 25 kHz, and the detection wavelength was set at 1000 nm.

Absolute Quantum Yield Measurements by Integrating Sphere
For the absolute QY measurements, the normal sample holder was removed, and the integrating sphere was mounted in FLS1000. The blank spectrum (purple line in Figure S15) was obtained by putting a blank into the sphere and making the emission scan (repeat 5 times) from 510 nm to 1600 nm with a 530 nm excitation. The sample spectrum (yellow line) was measured by replacing the blank with a dilute $Au_{20}Ag_{32}$ solution (0.1 OD at 530 nm) and repeating the emission (repeat 5 times) scan from 510 nm to 1600 nm with a 530 nm excitation. The PLQY is calculated by eq.:

$$\eta = \frac{L_{sample}}{E_{blank} - E_{sample}}$$

where E_{blank} is the area under the purple line between 510 nm and 541 nm (see Figure S15), E_{sample} is the area under the yellow line between 510 nm and 541 nm, L_{sample} is the area under the yellow line between 790 nm and 1400 nm.

Relative Quantum Yield Determination
The relative quantum yield (Φ_S) of the sample is calculated by using:

$$\phi_S = \phi_R \left(\frac{I_S}{I_R}\right)\left(\frac{1-10^{-A_R}}{1-10^{-A_S}}\right)\left(\frac{n_S}{n_R}\right)^2$$

In this context, $\mathbf{\Phi_R}$ denotes the quantum yield of the reference (standard), **I** represent the integrated photoluminescence (PL) intensity, **A** corresponds to the absorbance of the solution at the excitation wavelength, **n** stands for the refractive index of the solvent, and the subscripts (**S** and **R**) distinguish between the sample and the reference, respectively.

Transient Absorption Spectroscopy
Transient absorption measurements were performed using a broadband pump-probe setup, which is pumped by a 1 kHz Ti:Sapphire laser system (Spitfire, Spectra-Physics). The pump wavelengths (365 nm) were generated by a commercial optical parametric amplifier (TOPAS-C, Light Conversion). For femtosecond TA measurements, the supercontinuum probe light is generated by focusing the fundamental pulse (800 nm) into a sapphire plate. The probe light is split into signal and reference beams. The pump-probe delay was controlled by a mechanical delay line. For nanosecond TA measurements, the probe light is generated with a fiber laser (Leukos), and the delay times up to microseconds are controlled by an electronic delay configuration. TA measurements were performed in toluene, and the optical density of solution was adjusted to ~0.2 OD (2 mm cuvette) at excitation wavelength. The polarization of pump and probe pulse was set to magic angle (54.7°) to measure the isotropy signal. The transient absorption spectra were further analyzed using the publicly available program Glotaran based on the statistical fitting package TIMP (*J. Stat. Soft.* **2012**, *49*, 1–22 ; *Biochim. Biophys. Acta - Bioenerg.* **2004**, *1657*, 82–104).

X-ray Crystallography
A specimen of $C_{288}H_{312}Ag_{32}Au_{20}Cl_{12}$, approximate dimensions 0.060 mm x 0.100 mm x 0.160 mm, was used for the X-ray crystallographic analysis. The X-ray intensity data were measured (λ = 1.54178 Å).

A total of 3420 frames were collected. The total exposure time was 28.00 hours. The frames were integrated with the Bruker SAINT software package using a narrow-frame algorithm. The integration of the data using a monoclinic unit cell yielded a total of 215232 reflections to a maximum θ angle of 50.48° (1.00 Å resolution), of which 18182 were independent (average redundancy 11.838, completeness = 99.3%, R_{int} = 8.21%, R_{sig} = 3.83%) and 14801 (81.40%) were greater than $2\sigma(F^2)$. The final cell constants of a = 41.057(3) Å, b = 21.4055(13) Å, c = 41.103(2) Å, β = 105.074(3)°, volume = 34880.(4) $Å^3$, are based upon the refinement of the XYZ-centroids of 9785 reflections above 20 σ(I) with 6.057° < 2θ < 94.69°. Data were corrected for absorption

effects using the Multi-Scan method (SADABS). The ratio of minimum to maximum apparent transmission was 0.205. The calculated minimum and maximum transmission coefficients (based on crystal size) are 0.0280 and 0.1369. The final anisotropic full-matrix least-squares refinement on F^2 with 914 variables converged at R1 = 8.16%, for the observed data and wR2 = 27.88% for all data. The goodness-of-fit was 1.880. The largest peak in the final difference electron density synthesis was 2.182 $e^-/Å^3$ and the largest hole was -1.219 $e^-/Å^3$ with an RMS deviation of 0.217 $e^-/Å^3$. On the basis of the final model, the calculated density was 2.207 g/cm^3 and F(000), 21312 e^-.

S1.3. Theoretical Methodology

The electronic structure and optical spectrum of the $Au_{20}Ag_{32}$ nanocluster were investigated using density functional theory (DFT) and time-dependent density functional theory plus tight binding (TDDFT+TB) calculations while maintaining the crystallographic bond distances.[1] The calculations were executed in the gas phase employing the BP86 exchange-correlation functional, which is a generalized gradient approximation (GGA) functional, with a double-ζ (DZ) basis set.[2,3] Scalar relativistic effects were incorporated through the Zeroth-order regular approximation (ZORA).[4,5] For these computations, a tighter SCF convergence was achieved by setting the criterion to 1×10^{-8}. The optical spectrum was generated by smoothing the vertical excitation energies with a 30 nm full-width half maximum (FWHM) Gaussian broadening. All computational work was performed using the 2021 version of the Amsterdam Density Functional (ADF2021) software package.[6,7]

S1.4. An Investigation of the Molecular Orbital Characteristics of the $Au_{20}Ag_{32}$ nanocluster

Analysis of the molecular orbital characteristics reveals several notable features (**Figure S17**). At first glance, the superatomic orbital patterns within the occupied orbitals can be qualitatively rationalized using atomic orbital overlaps, such as p orbital interactions (**Figure S17**). Therefore, the orbitals near the HOMO-LUMO region exhibit apparent analogies to familiar bonding patterns, including σ* antibonding character at the HOMO (arising from an antibonding interaction of two superatomic P orbitals, similar to the antibonding interactions between two atomic p orbitals in a diatomic molecule), nonbonding character for HOMO-1 and HOMO-4 (which does not have a direct analog in diatomic molecules), π bonding character for HOMO-2, and π^* antibonding character for HOMO-3. While these orbital overlaps provide useful insights, they also suggest an effective separation of the molecular orbitals between the two halves of the cluster, imparting a dimer-like character to the orbital arrangement. This is most notable because of the presence of nonbonding orbitals, which indicate a low degree of coupling between the halves.

On the other hand, virtual orbitals exhibit distinct features compared to the occupied orbitals. Notably, the LUMO shows σ bonding character that can be associated with the overlap of two d_{z^2} type orbitals. However, at higher energies, the virtual orbitals are formed from intricate combinations of d orbitals, with overlap geometries and spatial orientations that deviate considerably from those typically observed in a nanowire or a nanorod system. The observed

deviations highlight that the electronic structure of this cluster is governed by orbital interactions beyond those captured by simple orbital overlap models.

S1.5. Hirshfeld Charge Distribution

We examined the atomic charge distribution within the nanocluster by analyzing the Hirshfeld charges of gold and silver atoms. **Figure S18** illustrates the charge distribution patterns of gold and silver atoms, categorized according to their structural origin (core vs. ligand-metal motifs that protect the core). Notably, both gold and silver atoms located in the core (**Figure S18a**) exhibit clear deviations from those in the ligand-metal motifs (**Figure S18b** and **S18c**). In particular, metal atoms in the motifs display a more positive character, with an average charge (q) of +0.21 for both gold and silver, compared to the corresponding atoms in the Au_8Ag_{15} core. Analysis of the Au_8Ag_{15} core reveals that the three bridging silver atoms exhibit substantially reduced positive charge, with an average value of +0.05. In contrast, the two gold atoms oriented toward these bridging silver atoms carry a slight negative charge (-0.03 average), while the remaining gold atoms are essentially charge neutral. The remaining silver atoms display intermediate positive charges in the range of +0.10 to +0.15. This distinct charge distribution suggests that the bridging silver atoms play an integral role in the core structure. Overall, these results highlight that metal atoms within the Au_8Ag_{15} core possess a distinguishable charge character compared to the metal atoms in the ligand-metal motifs.

S2. SUPPORTING FIGURES

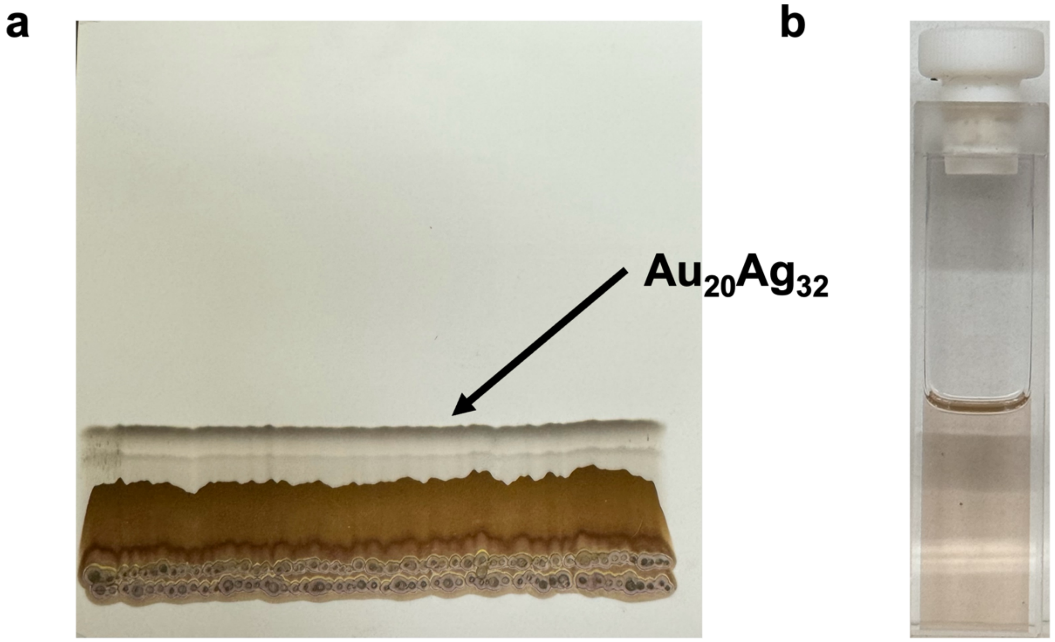

Figure S1. (a) TLC separation of $Au_{20}Ag_{32}$ from the product mixture. The top dark band is our product of interest (b) a concentrated $Au_{20}Ag_{32}$ solution in toluene.

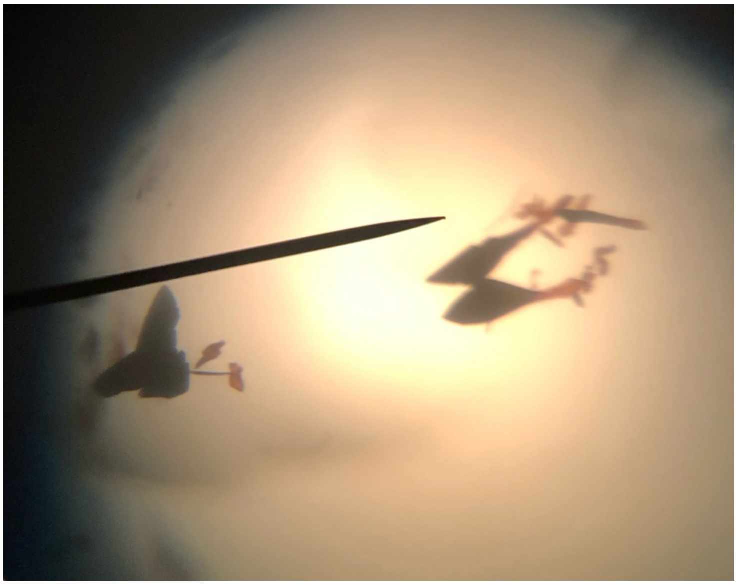

Figure S2. Optical microscopic image of $Au_{20}Ag_{32}$ crystals.

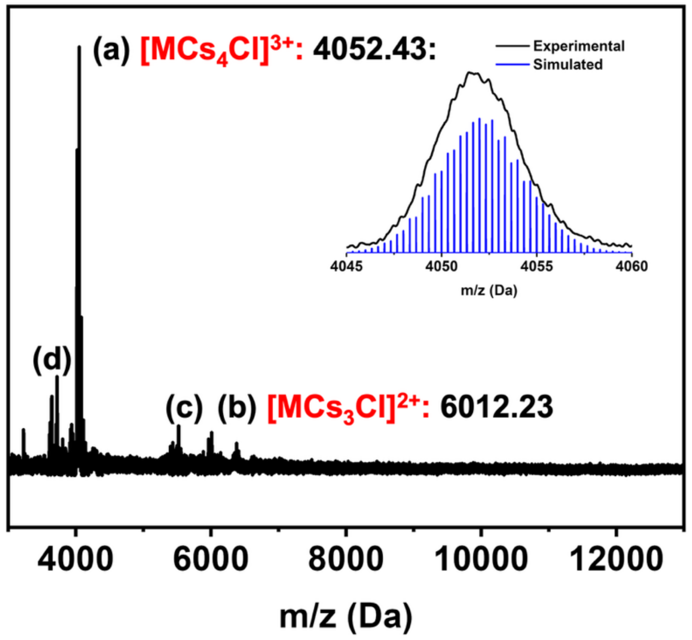

Figure S3. ESI-MS spectrum of $Au_{20}Ag_{32}$ in the presence of CsOAc in DCM (inset: experimental isotope pattern (black curve) vs the simulated one (blue bars). (a) and (b) are the principal MS peaks, while (c) and (d) are fragments formed under MS conditions with formula $[M]^{2+}$ – $Au(R)_2$ – Cl and . $[M]^{3+}$ – Ag(R), respectively

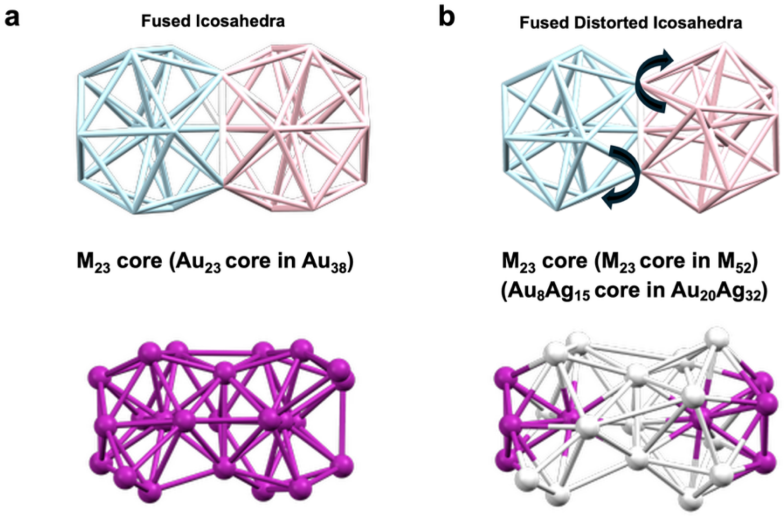

Figure S4. a) Face-fused Au_{23} biicosahedral core in $Au_{38}(PET)_{24}$ (note: the Au_{23} biicosahedron becomes distorted in Au_{38}(2,4-DMBT)$_{24}$, see Y. Li et al (***Nanoscale*** 2020, 12, 9423–9429), and b) the distorted M_{23} biicosahedral core in $Au_{20}Ag_{32}$ (this work).

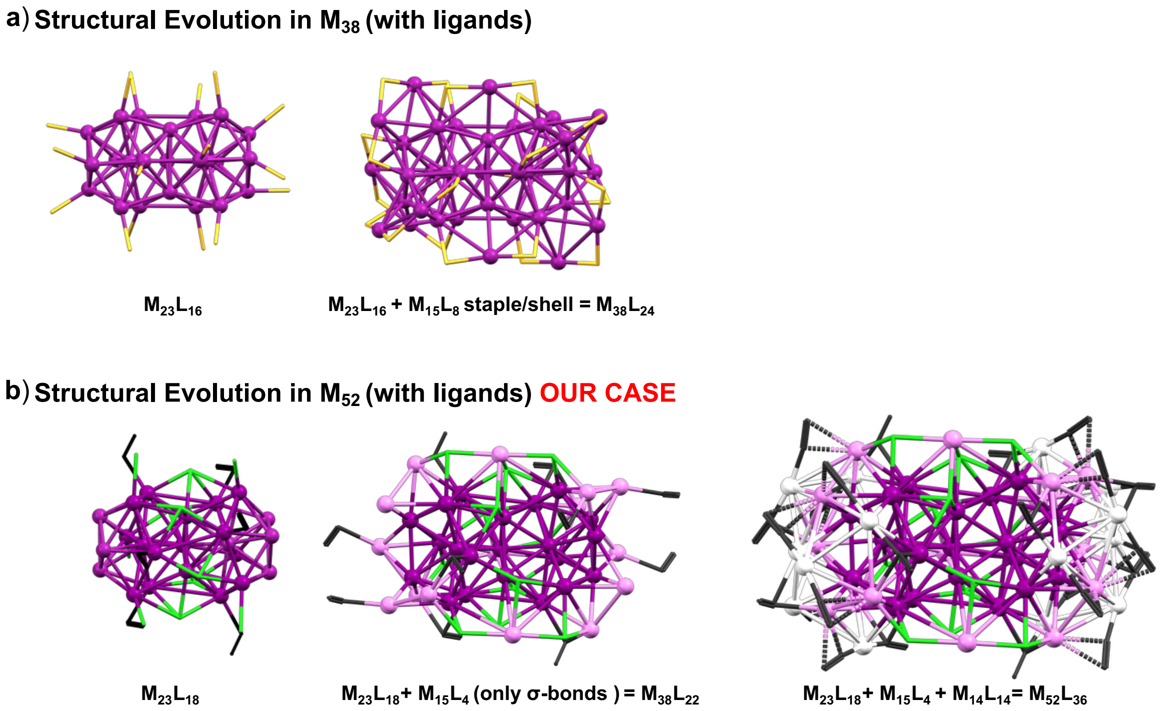

Figure S5. Comparison between the $Au_{38}(SR)_{24}$ (panel a, ref.[8]) and the $Au_{38}L_{22}$ partial structure in our M_{52} case (panel b).

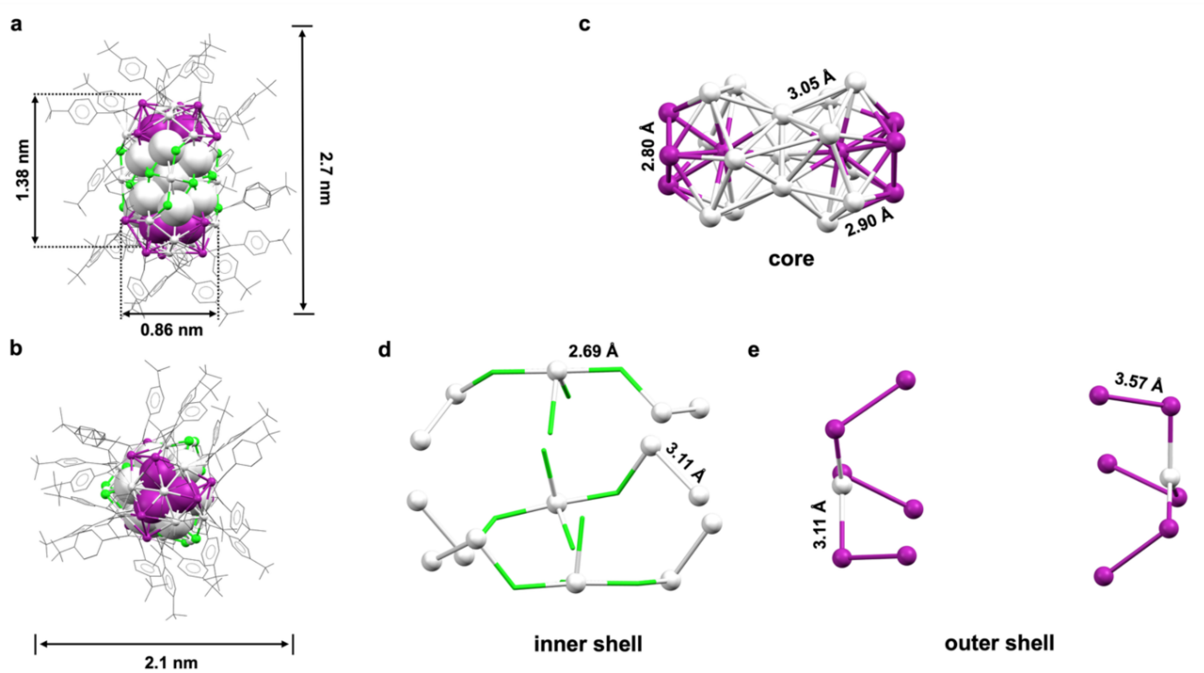

Figure S6. Dimensions of the $Au_{20}Ag_{32}$ NC (a) in the absence of and (b) presence of organic ligands. Average bond-lengths of $Au_{20}Ag_{32}$ in the (c) core, (d) shell at the belly of the M_{23} rod, (e) on the ends of the rod.

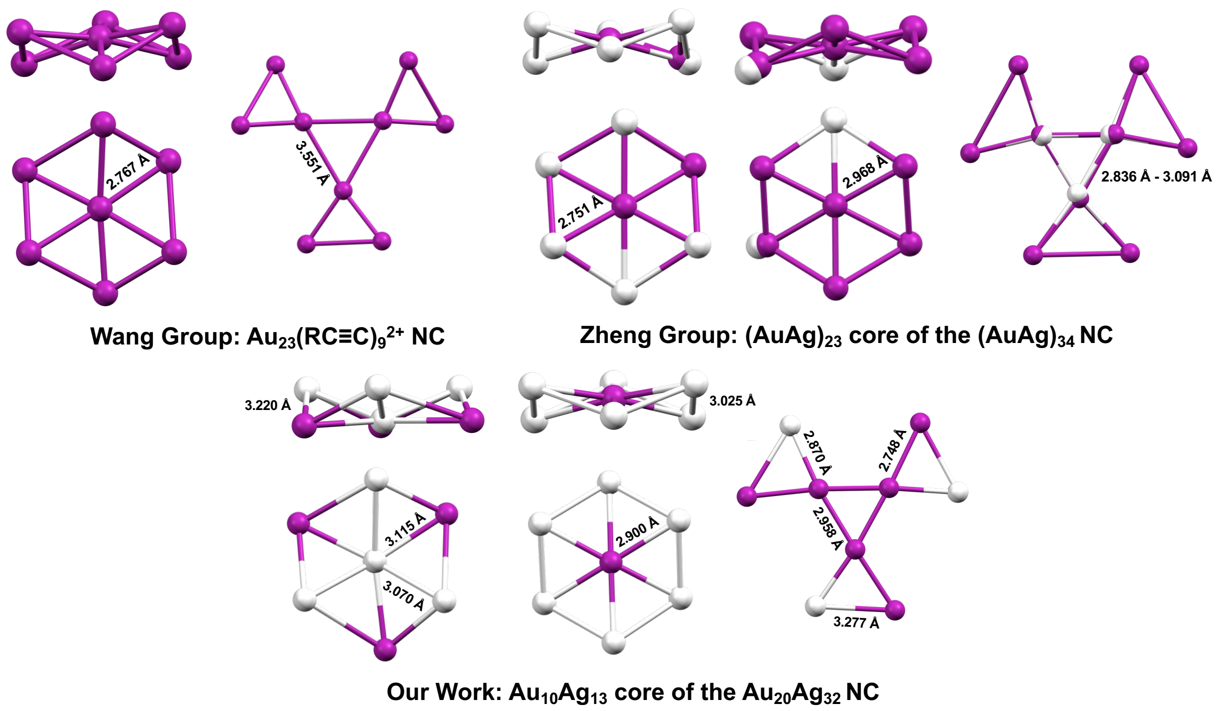

Figure S7. Bond-length comparison of M_7 rings in $Au_{20}Ag_{32}$ vs other reports[9,10] (through the M_{23}-dumbbell view).

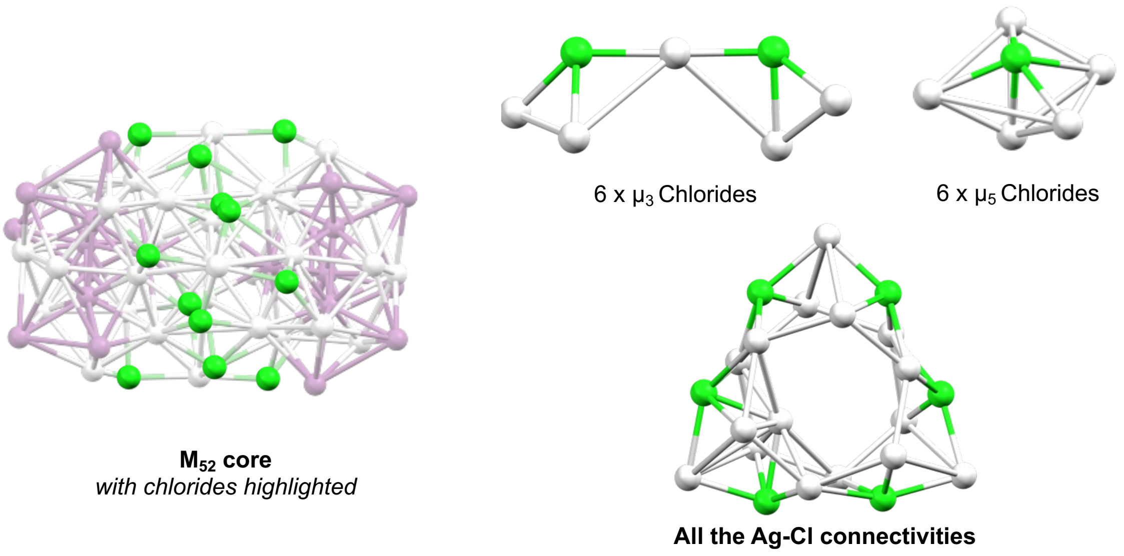

Figure S8. Different binding modes of Cl^- (green) ligands present in the NC, along with their spatial distribution on the NC.

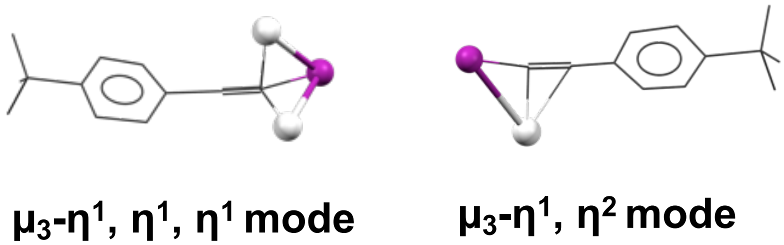

Figure S9. Two types of alkyne binding modes present in $Au_{20}Ag_{32}$ (purple balls: Au, grey balls: Ag).

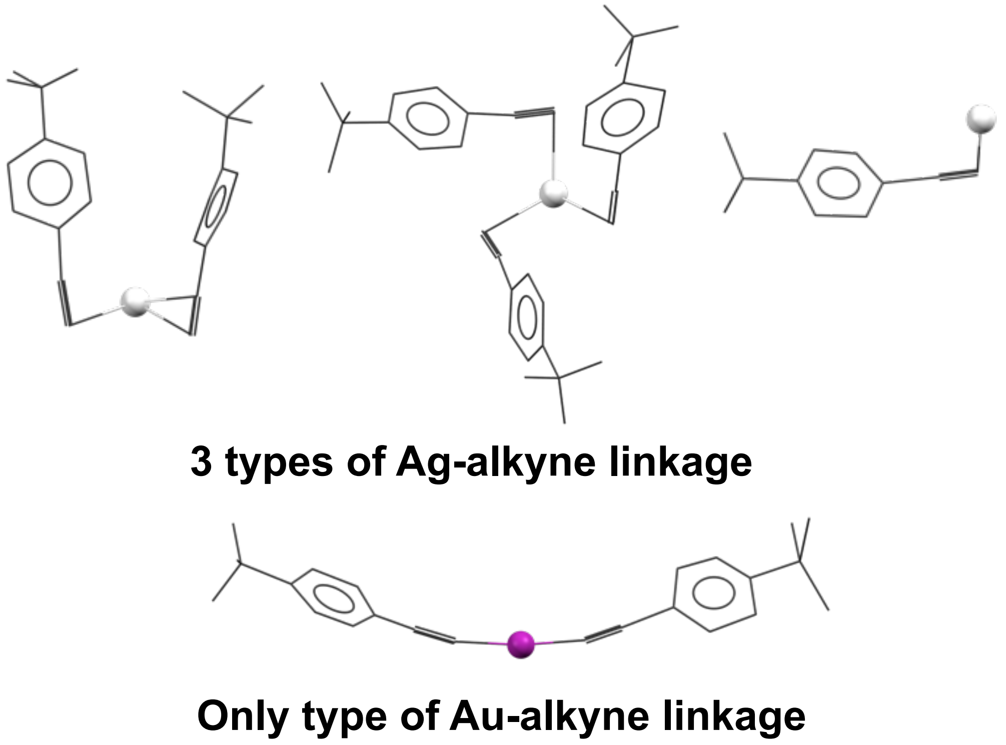

Figure S10. Various metal-alkyne linkages present in $Au_{20}Ag_{32}$.

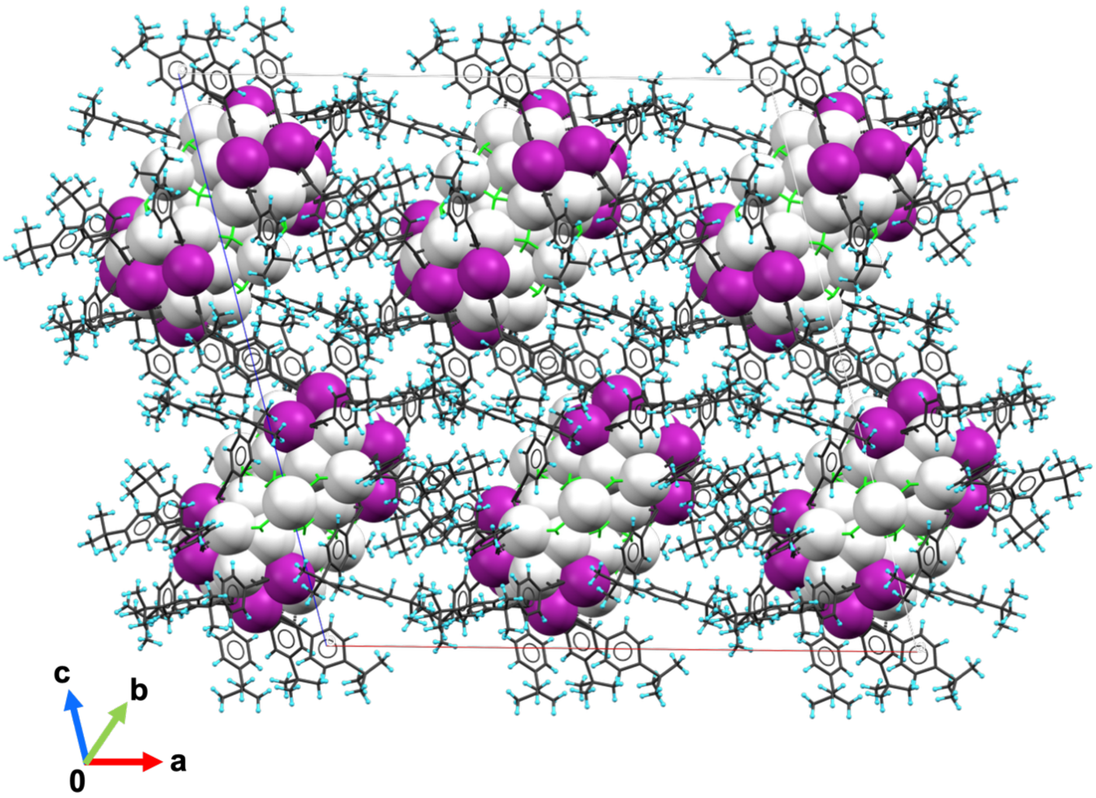

Figure S11. Crystal packing of M_{52} NCs following an ABAB pattern along the c-axis.

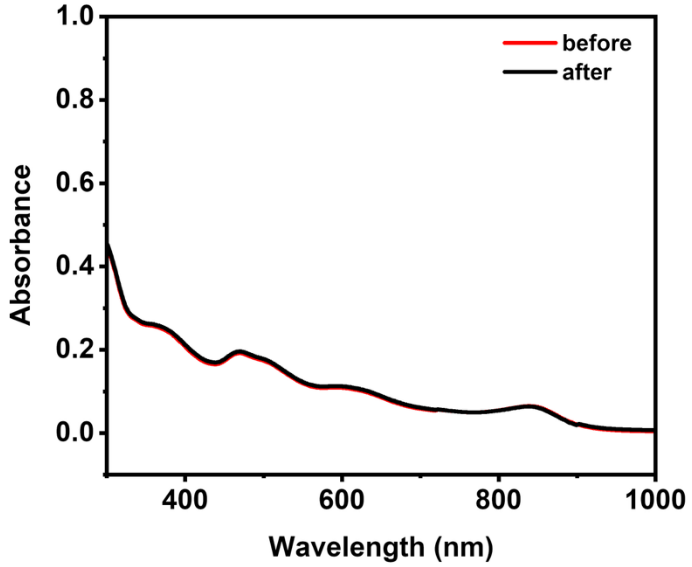

Figure S12. Stability of $Au_{20}Ag_{32}$ evaluated by comparing the UV-vis-NIR spectrum before and after storage in air for 30 days.

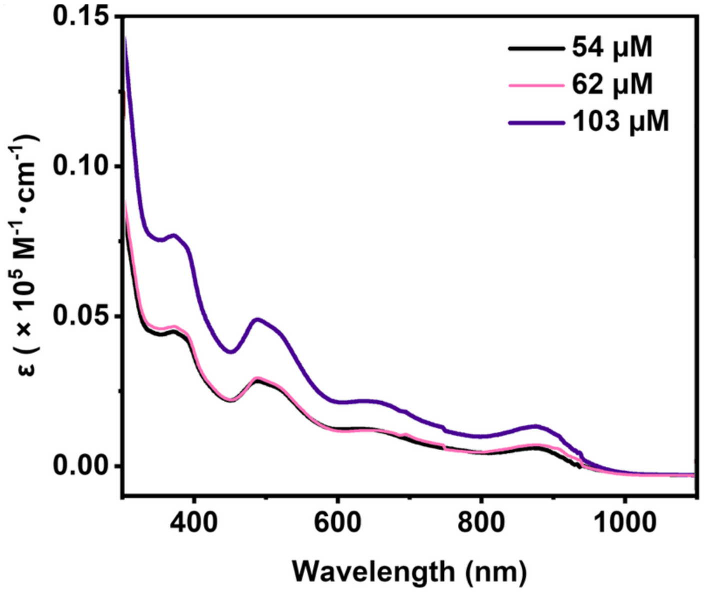

Figure S13. Absorptivity of $Au_{20}Ag_{32}$ under various concentrations (for example, absorption coefficient at 835 nm is 1.18×10^3 M^{-1} cm^{-1}).

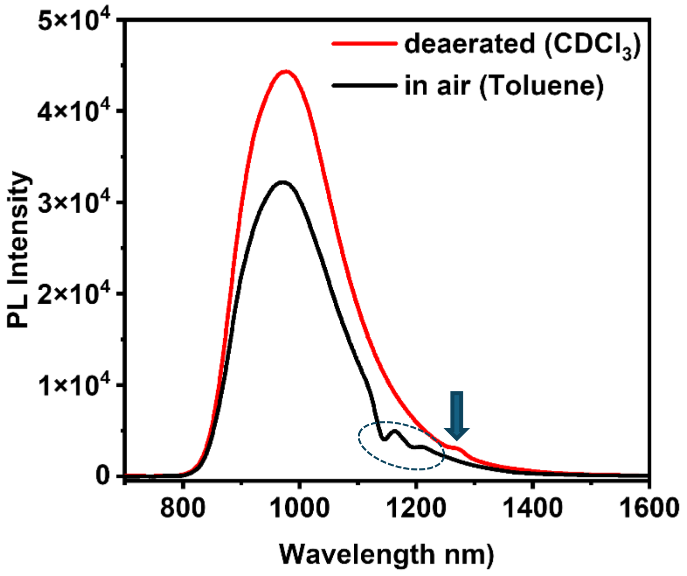

Figure S14. PL spectra of $Au_{20}Ag_{32}$ in degassed $CDCl_3$ (red) and in toluene (black). For PL measurements: excitation at 375 nm, slit width 8 nm, and emission slit 8 nm. Note: the region around 1150 -1220 nm (circled by dashed line) is distorted by toluene vibrational absorption, and the weak peak at 1275 nm is the singlet oxygen phosphorescence.

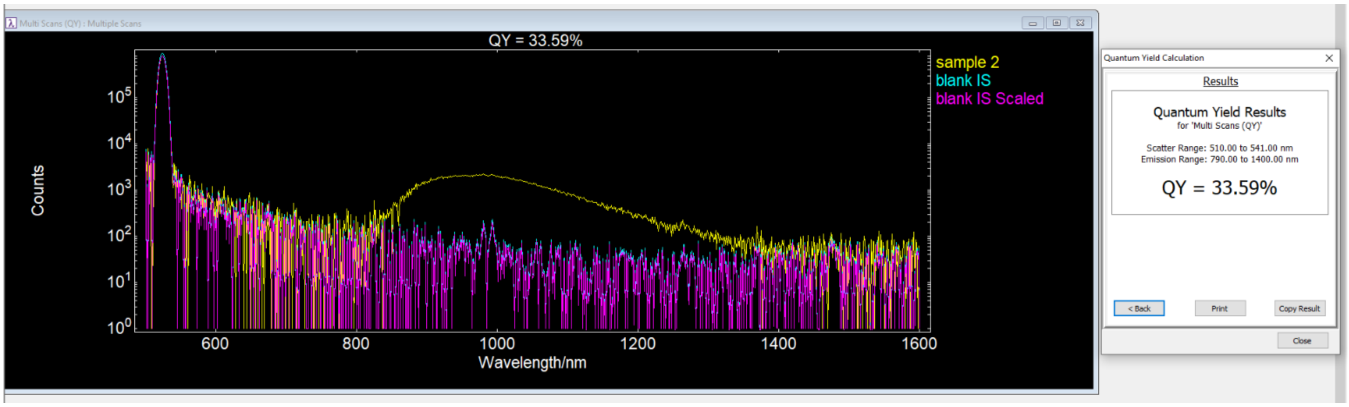

Figure S15. PLQY of $Au_{20}Ag_{32}$ in degassed $CDCl_3$ at room temperature measured by an integrating sphere (see Experimental for details). Note: y-axis is on a logarithmic scale.

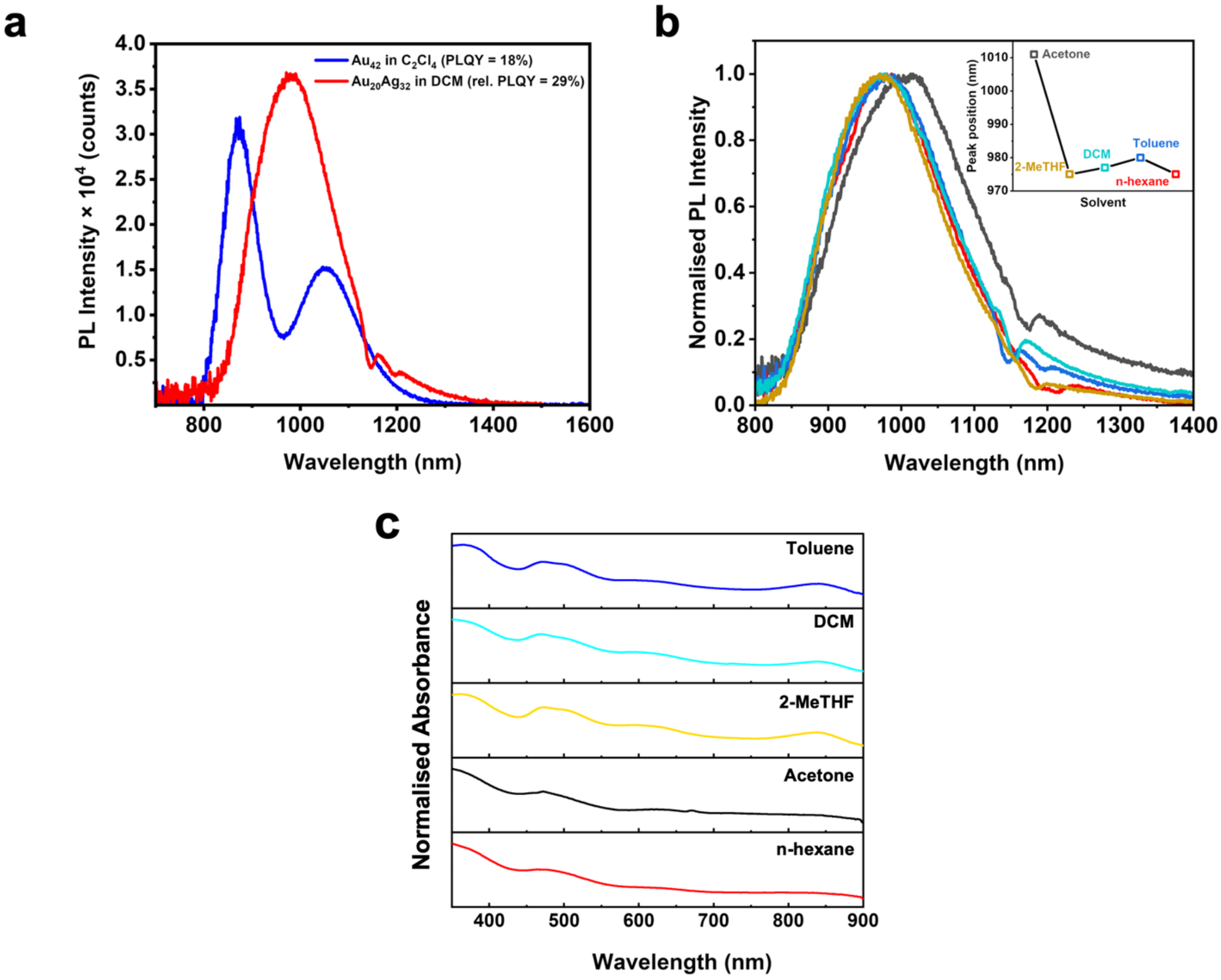

Figure S16. (a) PLQY of $Au_{20}Ag_{32}$ measured using relative method. Au_{42} NC in C_2Cl_4 is used as a reference, see L. Luo et. al. (***J. Am. Chem. Soc.*** 2024, 146, 27993–27997); (b) Normalized PL spectra of $Au_{20}Ag_{32}$ in different solvents. For PL measurements: excitation at 375 nm, slit width 8 nm, and emission slit 8 nm; inset: PL peak position of $Au_{20}Ag_{32}$ in acetone (black), chloroform (green), 2-methyltetrahydrofuran (gold), toluene (blue) and hexane (red) and (c) their corresponding normalized absorbance.

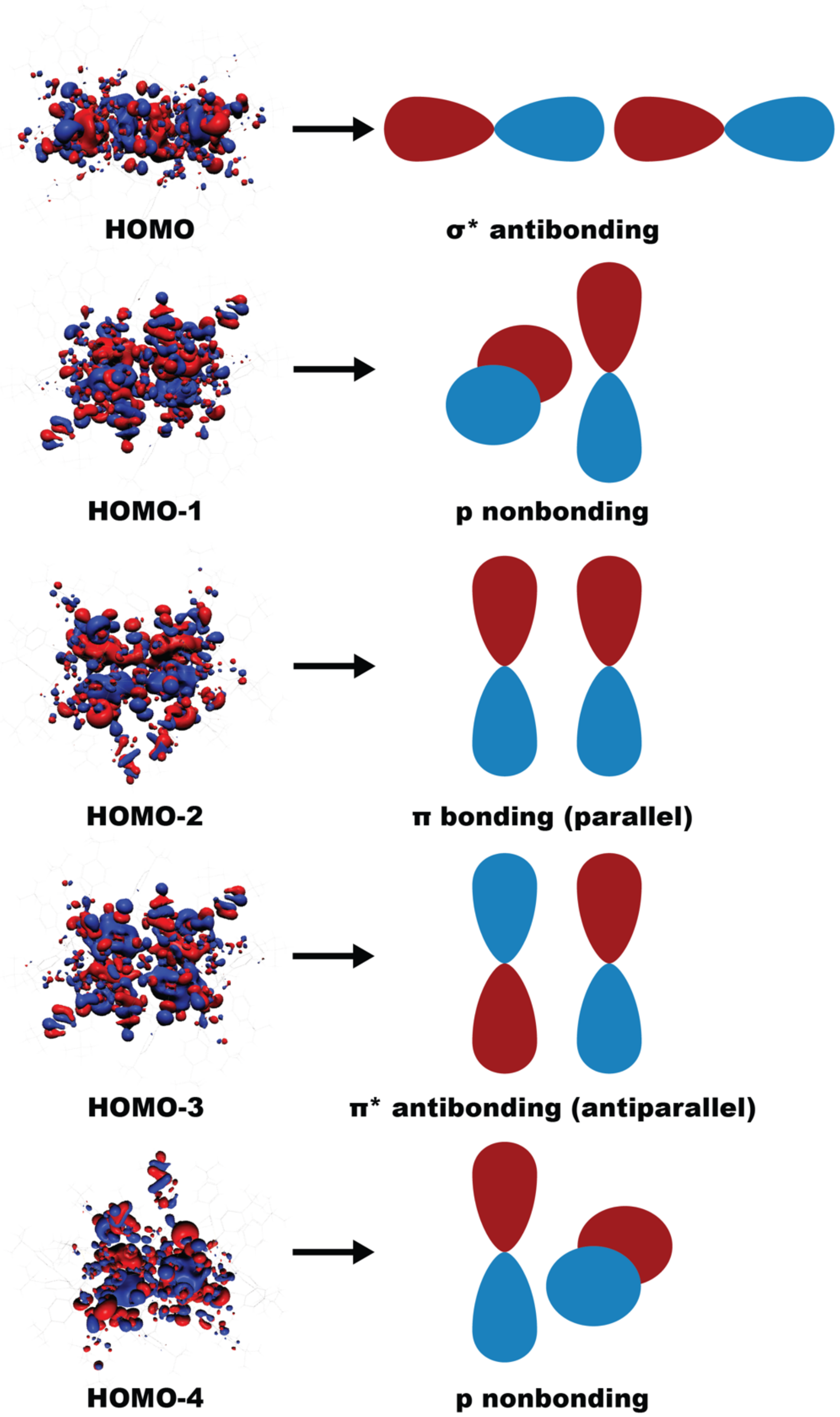

Figure S17. Selected occupied molecular orbitals of the $Au_{20}Ag_{32}$ nanocluster and their corresponding P (or *p*) orbital overlaps (at the BP86/DZ level of theory).

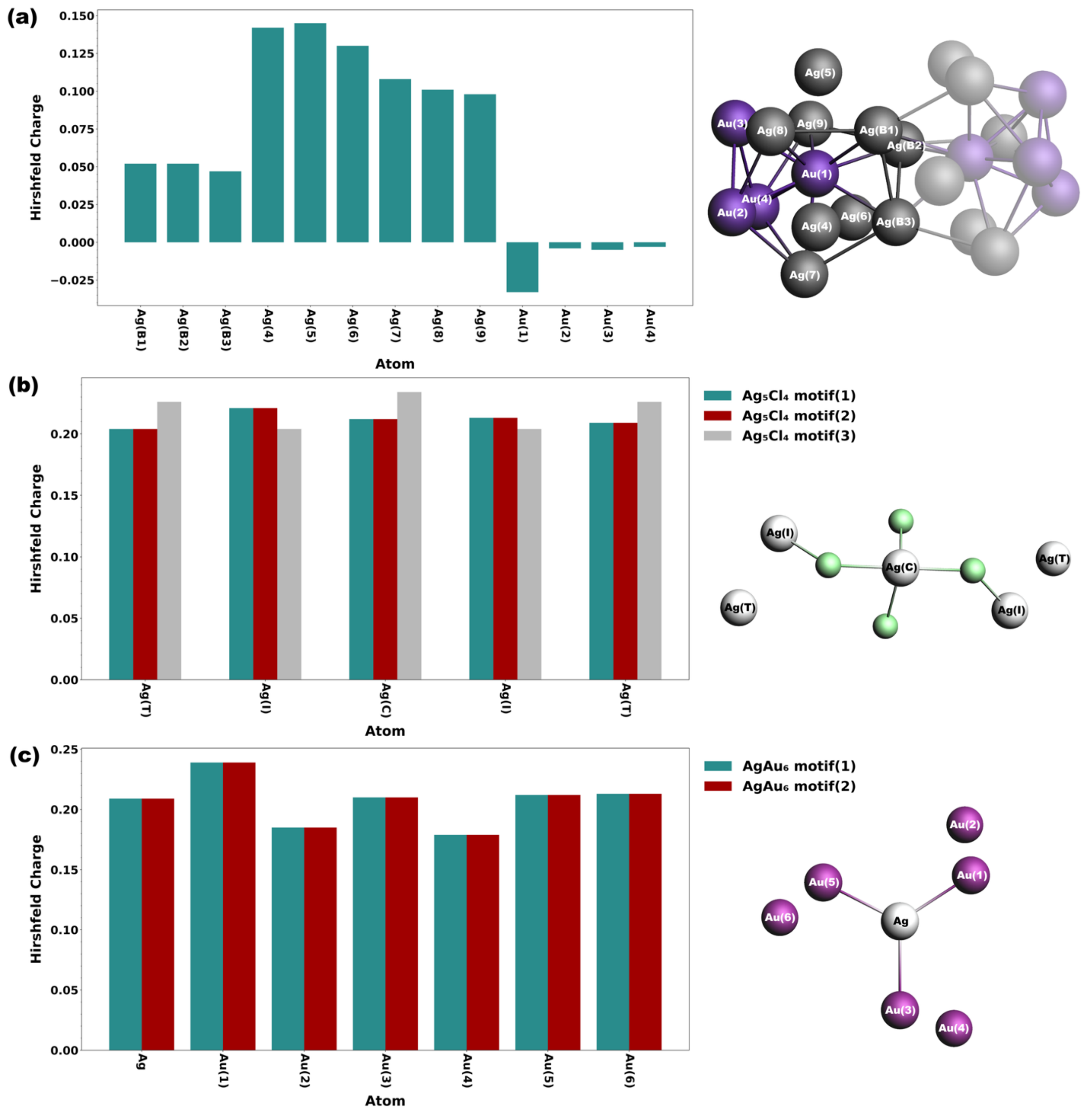

Figure S18. Comparative analysis of the Hirshfeld charge distribution for (a) Au_8Ag_{15} core (B1 - B3 denote the three bridging silver atoms), (b) Ag_5Cl_4 motif (C: center, I: intermediate, and T: terminal sites), and (c) $AgAu_6$ motifs. Color code: dark purple = core Au atoms, purple = Au atoms in the motifs, dark gray = core Ag atoms, gray = Ag atoms in the motifs.

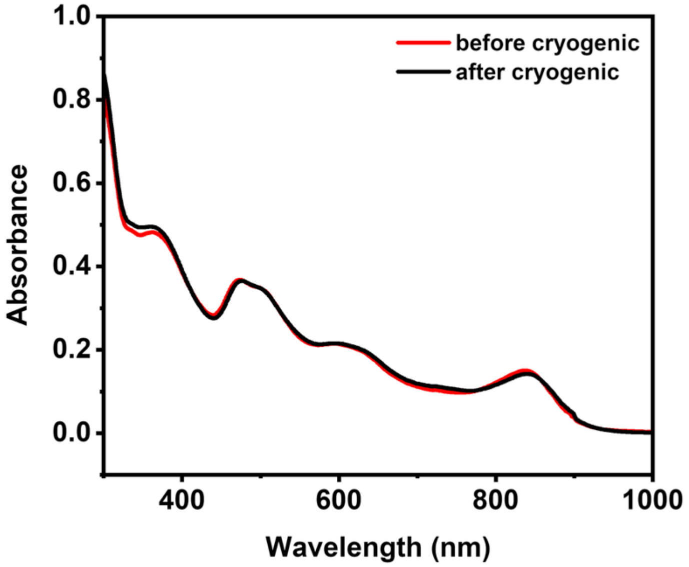

Figure S19. UV-vis-NIR spectrum of $Au_{20}Ag_{32}$ before (red) and after (black) cryogenic measurements.

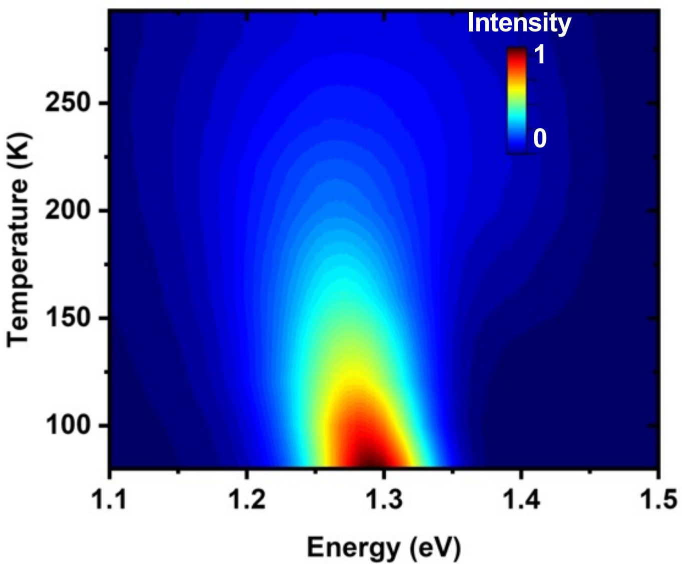

Figure S20. Temperature-dependent normalized emission map of $Au_{20}Ag_{32}$ in 2-MeTHF. The PL peak first redshifts and then blueshifts, indicating TADF.

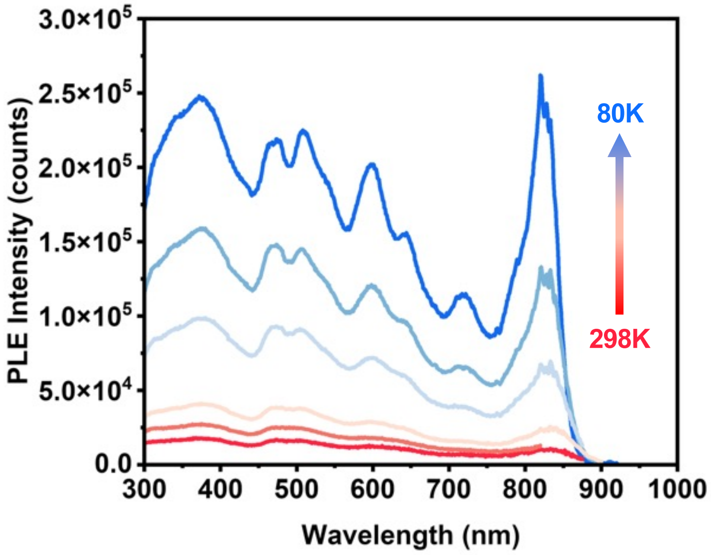

Figure S21. Temperature-dependent PL excitation spectra (as collected) of $Au_{20}Ag_{32}$ in 2-MeTHF.

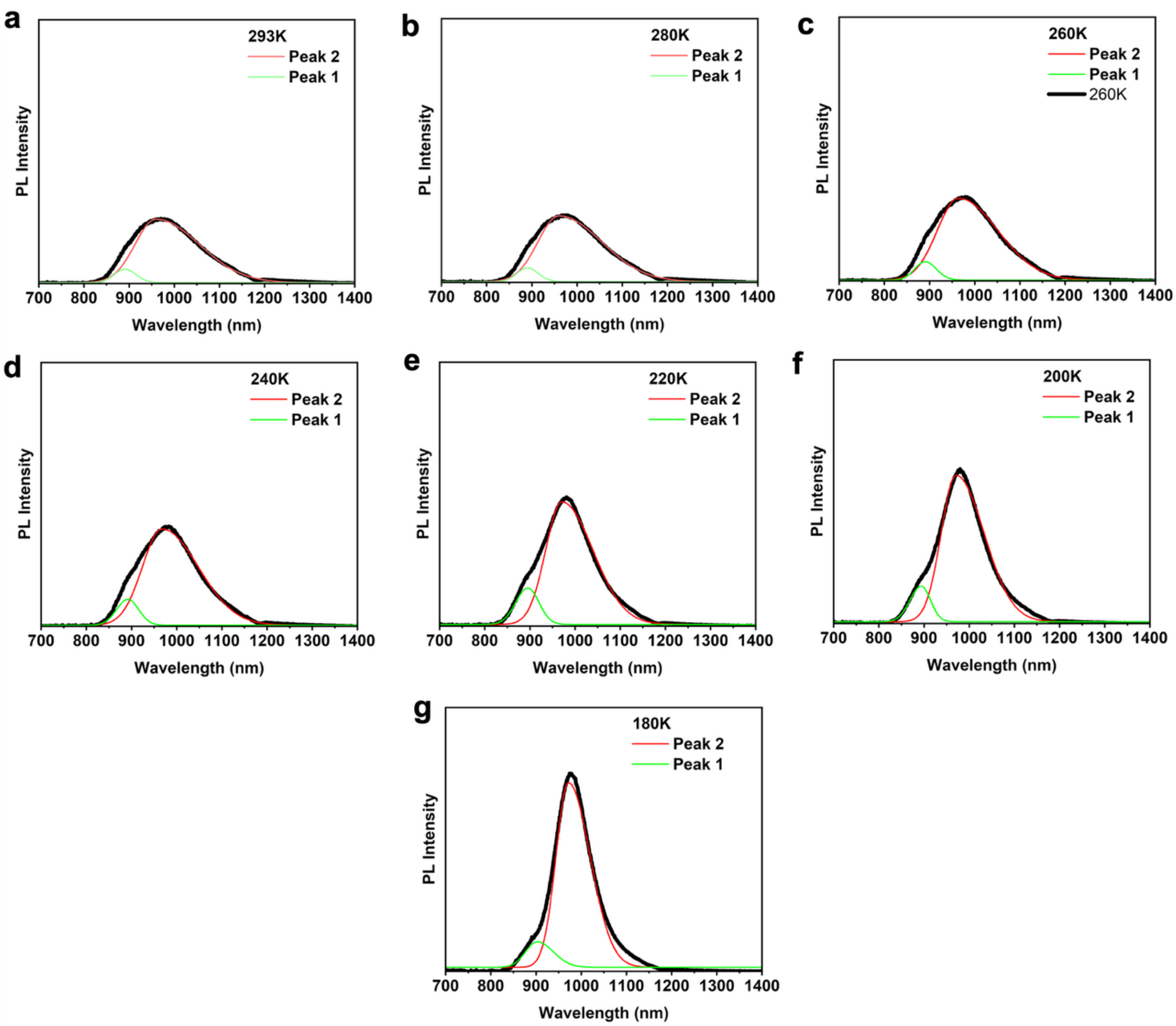

Figure S22. PL spectral peak deconvolution of $Au_{20}Ag_{32}$ at (a) 293K, (b) 280K, (c) 260K, (d) 240K, (e) 220K, (f) 200K, and (g) 180K, using pseudo-Voigt fitting. The green curves show the contribution from the shoulder band, whereas the red curves show the contribution from the main peak.

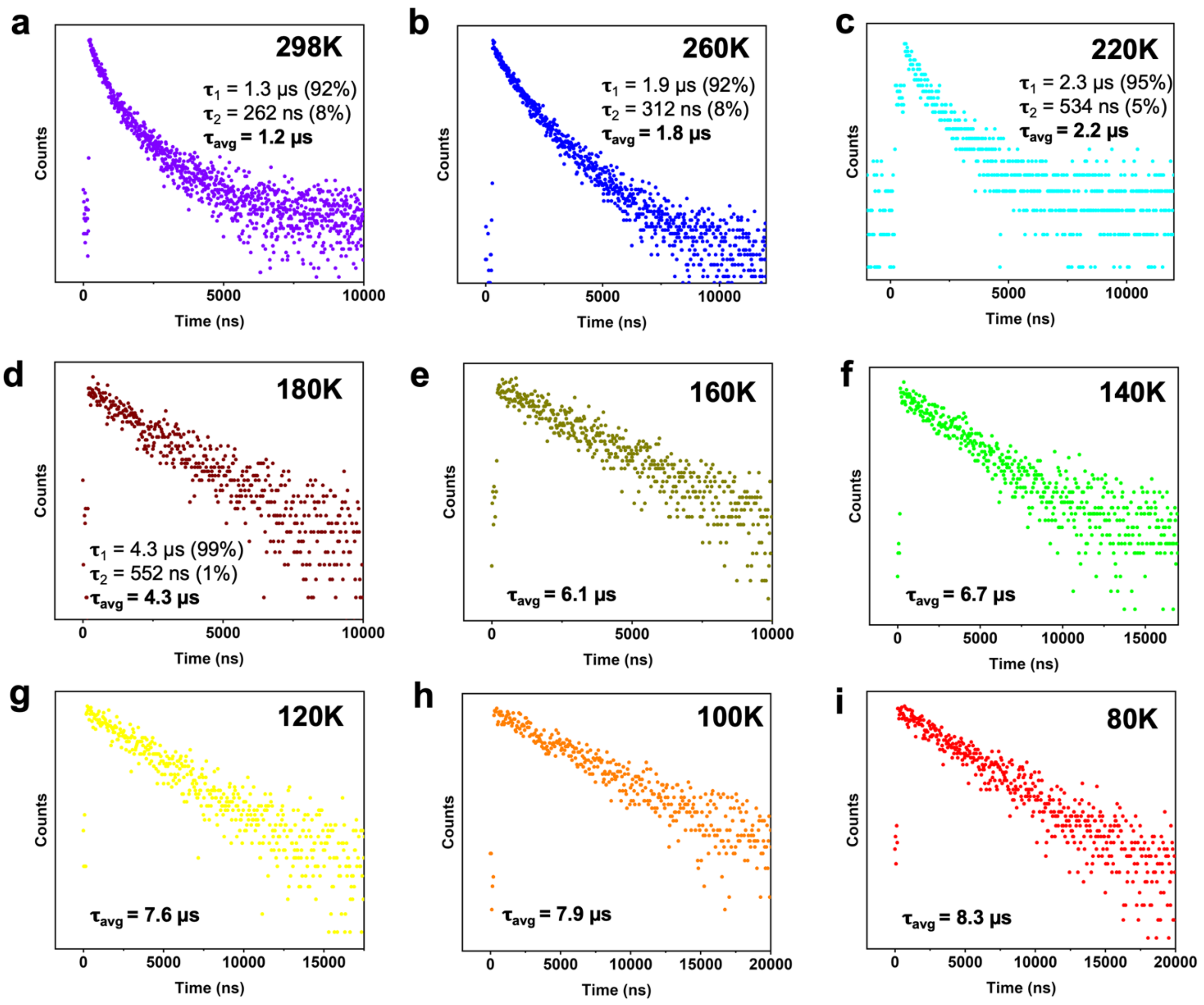

Figure S23. PL decay profiles of $Au_{20}Ag_{32}$ in 2Me-THF at (a) 293K, (b) 260K, (c) 220K, (d) 180K, (e) 160K, (f) 140K, (g) 120K, (h) 100K, and (i) 80K (detection at 1000 nm, MCS mode, see Experimental).

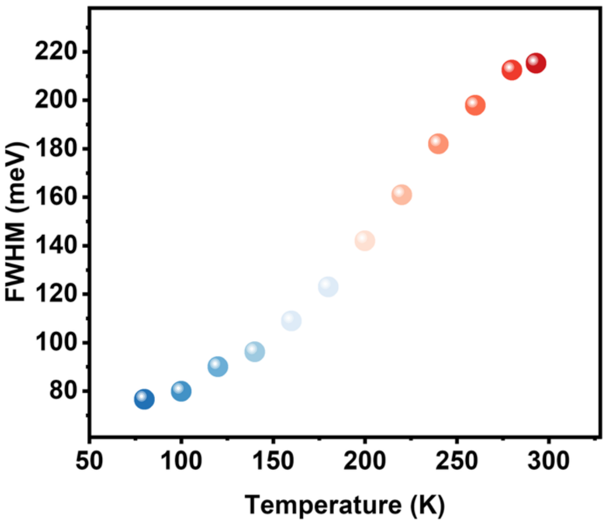

Figure S24. FWHM of the PL peak of $Au_{20}Ag_{32}$ in 2-MeTHF as a function of temperature.

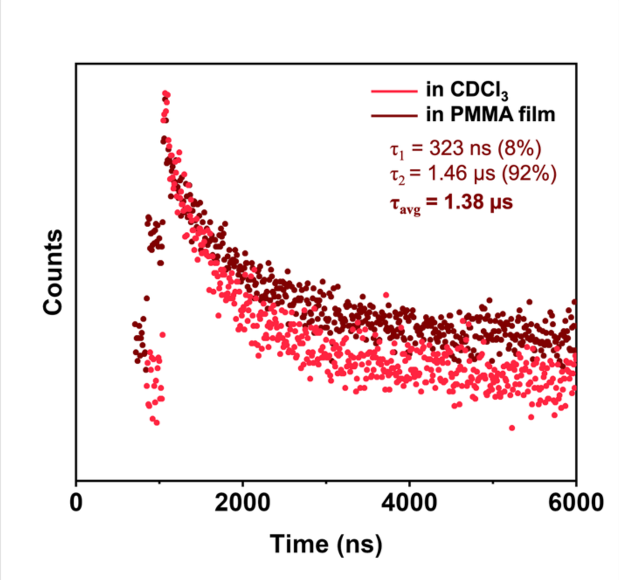

Figure S25. PL lifetime decay profiles of $Au_{20}Ag_{32}$ dissolved in $CDCl_3$ (red) and embedded in PMMA matrix (brown). Data were measured using $CDCl_3$ solutions of the NC (to avoid solvent re-absorption in the NIR). Inset: exponential fitting values for PL decay of $Au_{20}Ag_{32}$ embedded in PMMA film.

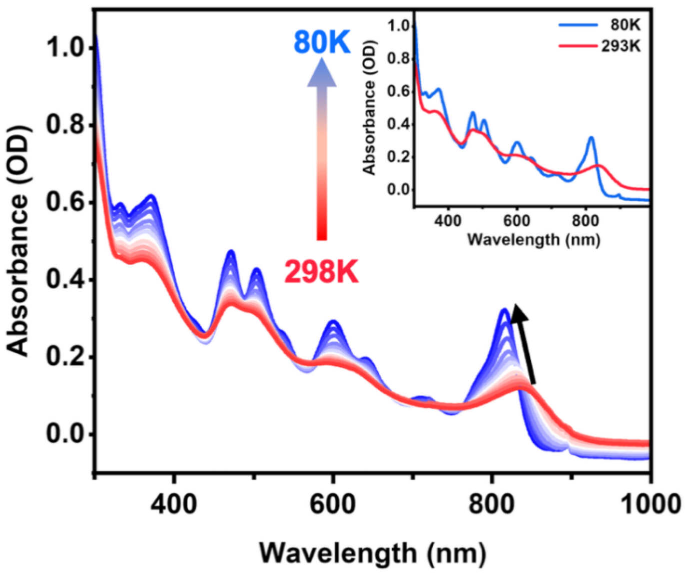

Figure S26. Temperature-dependent UV-vis-NIR spectra of $Au_{20}Ag_{32}$ in 2-MeTHF. Inset: magnified spectra of 298K and 80K cases.

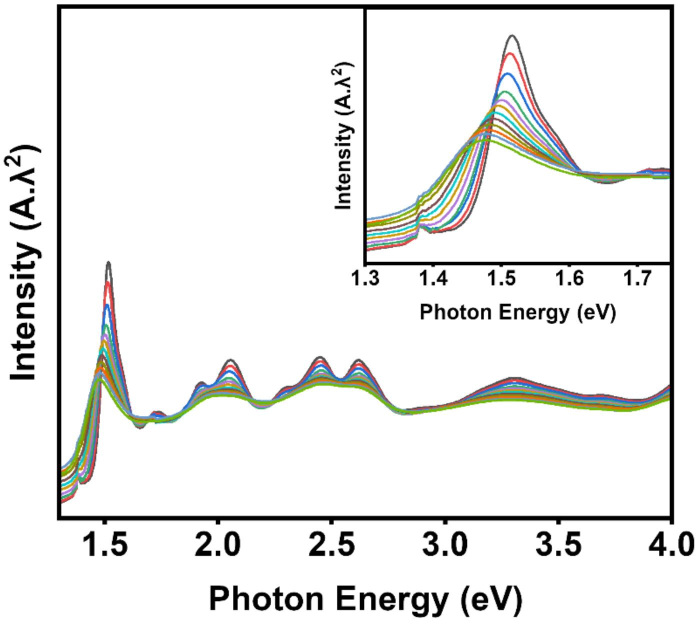

Figure S27. Temperature-dependent UV-vis-NIR spectra of $Au_{20}Ag_{32}$ in 2-MeTHF (photon-energy scale). Inset: magnified spectra of the lowest-energy peak to obtain E_g values.

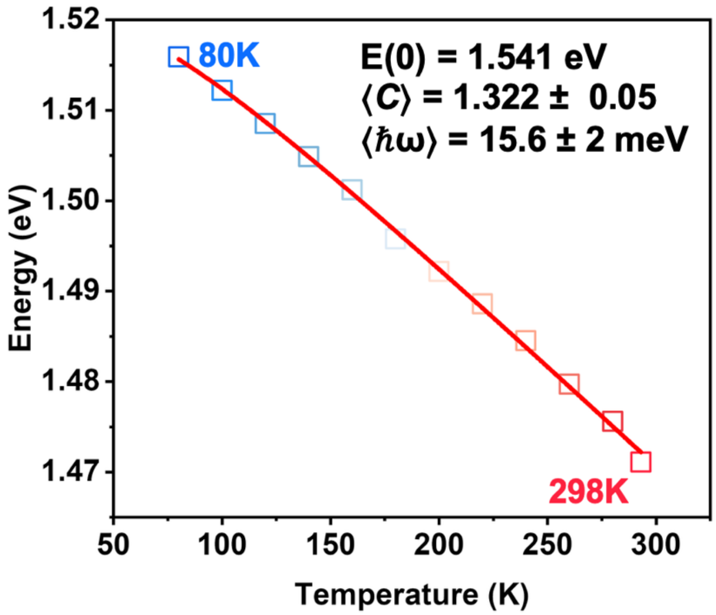

Figure S28. Temperature-dependent trend and fitting result (using the O'Donnell-Chen model) of the absorption maxima for the lowest-energy peak of $Au_{20}Ag_{32}$ in 2-MeTHF.

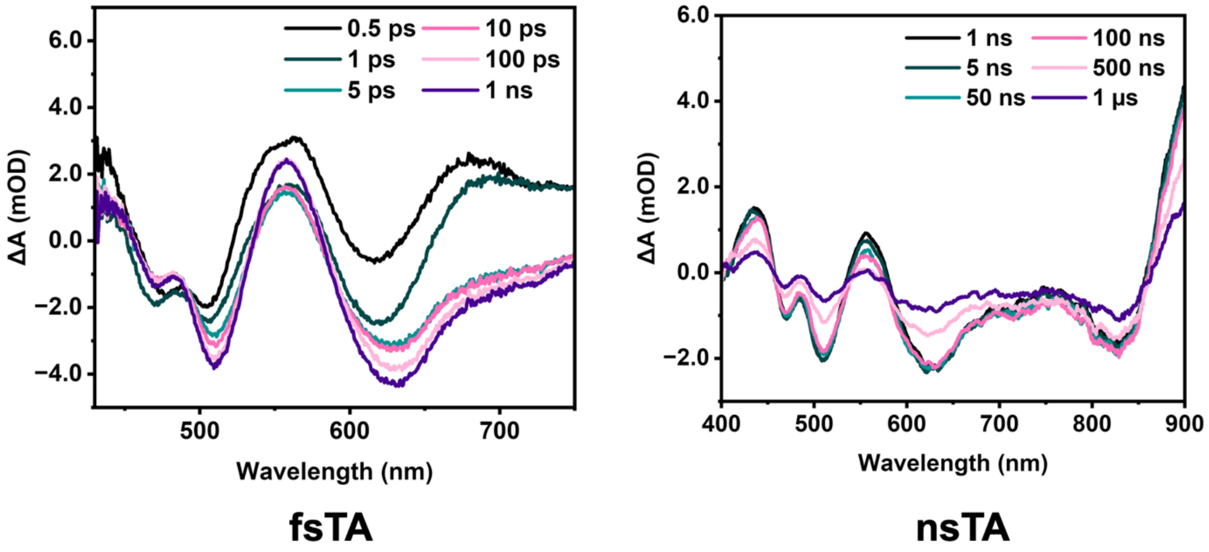

Figure S29. fs-TA and ns-TA spectra of $Au_{20}Ag_{32}$ in toluene at different time-delays.

S3. SUPPORTING TABLES

Table S1. Major NC product using various reaction conditions

Reaction Conditions (at 293K)			Products
Ag precursor	Au:Ag ratio	Ripening time	
$Ag(CH_3CN)_4{\cdot}BF_4$	3:1	-	Au NCs
$Ag(CH_3CN)_4{\cdot}BF_4$	2:1	-	$Au_{24}Ag_{20}$
$Ag(CH_3CN)_4{\cdot}BF_4$	2:1	1 day	$Au_{24}Ag_{20}$
$Ag(CH_3CN)_4{\cdot}BF_4$	2:1	3 days	$Au_{24}Ag_{20}$ + $\mathbf{Au_{20}Ag_{32}}$ (trace)
$Ag(CH_3CN)_4{\cdot}BF_4$	2:1	1 week	$Au_{24}Ag_{20}$ + $\mathbf{Au_{20}Ag_{32}}$ (~10%)
$Ag(CH_3CN)_4{\cdot}BF_4$	1:1	-	$Au_{24}Ag_{20}$ + $Au_{43}Ag_{38}$
$AgBF_4$/ $AgCH_3COO/AgNO_3$	-	-	$Au_{24}Ag_{20}$ + $Au_{43}Ag_{38}$

Table S2. Data collection details for $Au_{20}Ag_{32}Cl_{12}(CCPh^tBu)_{24}$.

Axis	dx/mm	2θ/°	ω/°	φ/°	χ/°	Width/°	Frames	Time/s	Wavelength/Å	Voltage/kV	Current/mA	Temperature/K
Omega	69.659	0.00	353.00	0.00	54.81	-1.00	180	10.00	1.54184	45	0.7	296
Phi	69.659	-20.00	210.00	0.00	54.81	-1.00	360	10.00	1.54184	45	0.7	296
Omega	69.659	-25.00	342.00	60.00	54.81	-1.00	180	10.00	1.54184	45	0.7	296
Omega	69.659	-40.00	320.00	120.00	54.81	-1.00	180	30.00	1.54184	45	0.7	296
Omega	69.659	-40.00	320.00	180.00	54.81	-1.00	180	30.00	1.54184	45	0.7	296
Omega	69.659	-65.00	302.00	240.00	54.81	-1.00	180	60.00	1.54184	45	0.7	296
Omega	69.659	-65.00	302.00	300.00	54.81	-1.00	180	60.00	1.54184	45	0.7	296
Omega	69.659	-65.00	302.00	0.00	54.81	-1.00	180	60.00	1.54184	45	0.7	296
Omega	69.659	-65.00	302.00	60.00	54.81	-1.00	180	60.00	1.54184	45	0.7	296
Phi	69.659	-20.00	290.00	0.00	54.81	-1.00	360	5.00	1.54184	45	0.7	296
Phi	69.659	-20.00	250.00	0.00	54.81	-1.00	360	10.00	1.54184	45	0.7	296
Omega	69.659	-25.00	342.00	90.00	54.81	-1.00	180	10.00	1.54184	45	0.7	296
Omega	69.659	-40.00	320.00	150.00	54.81	-1.00	180	30.00	1.54184	45	0.7	296
Omega	69.659	-40.00	320.00	210.00	54.81	-1.00	180	30.00	1.54184	45	0.7	296
Omega	69.659	-65.00	302.00	270.00	54.81	-1.00	180	60.00	1.54184	45	0.7	296
Omega	69.659	-65.00	302.00	330.00	54.81	-1.00	180	60.00	1.54184	45	0.7	296

Table S3. Sample and crystal data for $Au_{20}Ag_{32}Cl_{12}(CCPh^tBu)_{24}$.

**Chemical formula**	$C_{288}H_{312}Ag_{32}Au_{20}Cl_{12}$	
**Formula weight**	11589.93 g/mol	
**Temperature**	250(2) K	
**Wavelength**	1.54178 Å	
**Crystal size**	0.060 x 0.100 x 0.160 mm	
**Crystal system**	monoclinic	
**Space group**	C 1 2/c 1	
**Unit cell dimensions**	a = 41.057(3) Å	α = 90°
	b = 21.4055(13) Å	β = 105.074(3)°
	c = 41.103(2) Å	γ = 90°
**Volume**	34880.(4) $Å^3$	
**Z**	4	
**Density (calculated)**	2.207 g/cm^3	
**Absorption coefficient**	30.520 mm^{-1}	
**F(000)**	21312	

Table S4. Data collection and structure refinement for $Au_{20}Ag_{32}Cl_{12}(CCPh^tBu)_{24}$.

**Theta range for data collection**	2.35 to 50.48°	
**Index ranges**	-37<=h<=41, -21<=k<=21, -41<=l<=40	
**Reflections collected**	215232	
**Independent reflections**	18182 [R(int) = 0.0821]	
**Coverage of independent reflections**	99.3%	
**Absorption correction**	Multi-Scan	
**Max. and min. transmission**	0.1369 and 0.0280	
**Refinement method**	Full-matrix least-squares on F^2	
**Refinement program**	SHELXL-2019/2 (Sheldrick, 2019)	
**Function minimized**	Σ w(F_o^2 - F_c^2)^2	
**Data / restraints / parameters**	18182 / 385 / 914	
**Goodness-of-fit on F^2**	1.880	
**Δ/σ_{max}**	0.001	
**Final R indices**	14801 data; I>2σ(I)	R1 = 0.0816, wR2 = 0.2739
	all data	R1 = 0.0945, wR2 = 0.2788
**Weighting scheme**	$w=1/[\sigma^2(F_o^2)+(0.1060P)^2+2.0000P]$ where $P=(F_o^2+2F_c^2)/3$	
**Largest diff. peak and hole**	2.182 and -1.219 $eÅ^{-3}$	
**R.M.S. deviation from mean**	0.217 $eÅ^{-3}$	

Table S5. Atomically precise metal nanoclusters with reported quantum yield of >1% and PL maxima in the NIR-Ib to NIR-II region, that is, 900-1700 nm.

NC	Exc. (nm)	Em peak (nm)	Solvent	QY (%)	Ref
$Au_{37}(TBBT)_{21}(TPP)_2$	380	1,152	$CDCl_3$	1.5	11
$Au_{25}(BM)_{18}$	375	1100	DCM	1.7	12
$Au_{44}(MBA)_{26}$	-	1080	H_2O	5.5	13
$CdAg_{24}(2,4\text{-DMBT})_{18}$	500	1045	$CDCl_3$*	1.7	15
$Au_{42}(PET)_{32}$	380	**DE:** 806, 1040	DCM	11.2	14
$Au_xAg_{25-x}(2,4\text{-DMBT})_{18}$	500	1050	$CDCl_3$*	5	15
$Ag_{25}(2,4\text{-DMBT})_{18}$	500	1010	$CDCl_3$*	3.5	15
$[Au_{25}(PPh_3)_{10}(PET)_5Cl_2]^{2+}$	-	990	DCM	8	16
**$Au_{20}Ag_{32}Cl_{12}(^tBuPA)_{24}$**	**525**	**980**	**$CDCl_3$***	**33**	**This work**
$Au_{10}Ag_2(2pyC{\equiv}C)_3(dppy)_6]^{5+}$	480	971	DCM	4.9	17
$[K_3Au_{25}(2\text{-Hmna})_9(mna)_6]^-$	-	970	H_2O	1.6	18
$Au_{76}(p\text{-MBT})_{42}$	400	970	Toluene	30	28
$[Au_{20}(CZ\text{-PrA})_{16}]^{2-}$	365	**DE:** 820, 940	DCM	6.3	19
$Au_{52}(TBBT)_{32}$	470	938	DCM	3.8	20
$PdAg_{24}(2,4\text{-DMBT})_{18}$	400	925	Acetonitrile	17	21
$Au_{52}(iPBT)_{32}$	470	922	DCM	14.7	20
$Au_{52}(PET)_{32}$	470	915	DCM	3.2	27
$[Au_{28}(p\text{-MBT})_{14}(Hdppa)_3]^{2+}$	540	920	DCM	12	22
$Au_{52}(EBT)_{32}$	470	916	DCM	11.1	20
$Au_{39}(PET)_{29}$	400	915	$CDCl_3$*	19	23
$AuAg_{24}(2,4\text{-DMBT})_{18}$	500	910	$CDCl_3$*	35	15
$[PtCdAu_{23}(PET)_{18}]^-$	700	902	-	2.2	24
$Au_{38}S_2(SAdm)_{20}$	500	900	Toluene	15	25
$Au_{52}(pMBT)_{32}$	470	900	DCM	18.3	20
$Au_{28}(^tBuPA)_{20}$	800	895	DCM	2.8	26

Notes:
Dark gray: **NIR-II window**
Light gray: **NIR-Ib window**
DE: Dual emission
Asterisk *: deaerated

Table S6. Important molecular orbital characteristics for the $Au_{20}Ag_{32}$ nanocluster at the BP86/DZ level of theory.

Molecular orbital	Primary characteristics
LUMO+8	Individual p_y, p_z, and s orbitals have a contribution on Au atoms (both p_y and p_z orbitals have contributions < 5.0 %, while s atomic orbitals have a contribution > 10.0 %); p_z and s atomic orbitals on Ag atoms (have contributions < 5.0 %)<br>**Metal-centered molecular orbital dominated by gold atomic orbital contributions**
LUMO+7	Individual p_y and s atomic orbitals have contributions on Au atoms (have contributions > 5.0 %); s atomic orbitals on Ag atoms (has a contribution < 5.0 %)<br>**Metal-centered molecular orbital dominated by gold atomic orbital contributions**
LUMO+1	Individual p_x, p_z, and s orbitals on Ag atoms (p_x and $p_z$atomic orbitals have a contribution < 5.0 %, while s orbitals have > 15.0 % contribution); p_x, p_y, p_z, and s orbitals on Au atoms (have contributions < 5.0 %); p_x, p_y, and p_z orbitals on Cl atoms (have contributions < 5.0 %)<br>**Metal-centered molecular orbital dominated by silver atomic orbital contributions**
LUMO	Individual p_x and p_z atomic orbitals on Au atoms (have contributions < 10.0 %); p_y and s orbitals on Ag atoms (p_y has a contribution < 5.0, while s atomic orbitals have a contribution > 10.0 %)<br>**Metal-centered molecular orbital dominated by gold atomic orbital contributions**
HOMO	Individual d_{xz} and s atomic orbitals have contributions on Au atoms (d_{xz} orbitals have a contribution < 5.0 %, while s orbitals have a contribution > 25.0 %); d_{yz} orbitals on Ag atoms (has a contribution < 5.0 %); p_x orbitals on Cl atoms (has a contribution < 5.0 %)<br>**Metal-centered molecular orbital dominated by gold atomic orbital contributions**
HOMO-1	Individual s atomic orbitals have contributions on Au atoms (has a contribution > 15.0 %); s atomic orbitals on Ag atoms (has a contribution < 5.0 %)<br>**Metal-centered molecular orbital dominated by gold atomic orbital contributions**
HOMO-2	Individual p_y and s atomic orbitals have contributions on Au atoms (p_y has a contribution < 10.0 %, while s atomic orbitals have a contribution > 10.0 %); d_{xz} and s atomic orbitals on Ag atoms (both have a contribution < 5.0 %); p_y orbitals on Cl atoms (has a contribution < 5.0 %)<br>**Metal-centered molecular orbital dominated by gold atomic orbital contributions**
HOMO-3	Individual s atomic orbitals have contributions on Au atoms (has a contribution > 15.0 %)<br>**Metal-centered molecular orbital dominated by gold atomic orbital contributions**
HOMO-4	Individual p_y and s atomic orbitals have contributions on Au atoms (p_y has a contribution > 5.0 %, while s atomic orbitals have a contribution > 15.0 %); p_x and s orbitals on Ag atoms (both have a contribution < 5.0 %); p_x and p_y orbitals on Cl atoms (have contributions < 5.0 %)<br>**Metal-centered molecular orbital dominated by gold atomic orbital contributions**

Table S7. Excitation energies, oscillator strengths, electronic transitions, and respective weights for the prominent absorption peaks of the $Au_{20}Ag_{32}$ nanocluster from TDDFT+TB calculations at the BP86/DZ level of theory.

Excitation energy	Oscillator strength (a.u.)	Transitions	Weight
1.25 eV (988.14 nm)	0.0299	HOMO-1 → LUMO	72.1 %
		HOMO → LUMO	25.0 %
1.32 eV (940.46 nm)	0.1808	HOMO → LUMO	52.0 %
		HOMO-1 → LUMO	25.3 %
1.53 eV (807.78 nm)	0.1516	HOMO-3 → LUMO+1	37.8 %
		HOMO-1 → LUMO+3	17.5 %
		HOMO-1 → LUMO+2	12.1 %
1.73 eV (715.60 nm)	0.0790	HOMO-3 → LUMO+4	26.8 %
		HOMO-1 → LUMO+5	19.9 %
		HOMO-2 → LUMO+4	16.5 %
		HOMO-4 → LUMO+3	11.3 %
2.01 eV (617.07 nm)	0.0979	HOMO-3 → LUMO+7	43.0 %
		HOMO-2 → LUMO+7	16.0 %
2.08 eV (594.84 nm)	0.0895	HOMO-4 → LUMO+8	54.4 %
2.58 eV (481.33 nm)	0.0549	HOMO-6 → LUMO+13	28.6 %

SUPPORTING REFERENCES

1 R. Rüger, E. van Lenthe, T. Heine, L. Visscher, *J. Chem. Phys.* **2016**, *144*, 184103.
2 A. D. Becke, *Phys. Rev. A* **1988**, *38*, 3098–3100.
3 J. P. Perdew, *Phys. Rev. B* **1986**, *33*, 8822–8824.
4 E. van Lenthe, E. J. Baerends, J. G. Snijders, *J. Chem. Phys.* **1993**, *99*, 4597–4610.
5 E. van Lenthe, A. Ehlers, E.-J. Baerends, *J. Chem. Phys.* **1999**, *110*, 8943–8953.
6 G. te Velde, F. M. Bickelhaupt, E. J. Baerends, C. Fonseca Guerra, S. Dr. van Gisbergen, J. G. Snijders, T. Ziegler, *J. Comp. Chem.* **2001**, *22*, 931–967.
7 ADF2021, SCM. Theoretical Chemistry; Vrije Universities: Amsterdam, 2021. http://www.scm.com (accessed 12 June 2025)
8 H. Qian, W. T. Eckenhoff, Y. Zhu, T. Pintauer, R. Jin, *J. Am. Chem. Soc.* **2010**, *132*, 8280–8281.
9 P. Yuan, R. Zhang, E. Selenius, P. Ruan, Y. Yao, Y. Zhou, S. Malola, H. Häkkinen, B. K. Teo, Y. Cao, N. Zheng, *Nat. Commun.* **2020**, *11*, 2229.
10 X.-K. Wan, S.-F. Yuan, Q. Tang, D. Jiang, Q.-M. Wang, *Angew. Chem. Int. Ed.* **2015**, *54*, 5977–5980.

(refs 11-28 for Table S4):
11 Y. Tan, K. Li, J. Xu, Q. Li, S. Yang, J. Chai, Y. Pei, D. Jia, M. Zhu, *Nanoscale* **2024**, *16*, 15663–15669.
12 Z. Liu, Y. Li, E. Kahng, S. Xue, X. Du, S. Li, R. Jin, *ACS Nano* **2022**, *16*, 18448–18458.
13 G. Yang, X. Pan, W. Feng, Q. Yao, F. Jiang, F. Du, X. Zhou, J. Xie, X. Yuan, *ACS Nano* **2023**, *17*, 15605–15614.
14 L. Luo, Z. Liu, X. Du, R. Jin, *J. Am. Chem. Soc.* **2022**, *144*, 19243–19247.
15 Z. Liu, M. Zhou, L. Luo, Y. Wang, E. Kahng, R. Jin, *J. Am. Chem. Soc.* **2023**, *145*, 19969–19981.
16 Q. Li, C. J. Zeman, Z. Ma, G. C. Schatz, X. W. Gu, *Small* **2021**, *17*, 2007992.
17 Z. Lei, Z.-J. Guan, X.-L. Pei, S.-F. Yuan, X.-K. Wan, J.-Y. Zhang, Q.-M. Wang, *Chem. – Eur. J.* **2016**, *22*, 11156–11160.
18 W.-D. Tian, W.-D. Si, S. Havenridge, C. Zhang, Z. Wang, C. M. Aikens, C.-H. Tung, D. Sun, *Sci. Bull.* **2024**, *69*, 40–48.
19 W.-D. Si, C. Zhang, M. Zhou, W.-D. Tian, Z. Wang, Q. Hu, K.-P. Song, L. Feng, X.-Q. Huang, Z.-Y. Gao, C.-H. Tung, D. Sun, *Sci. Adv.* **2023**, *9*, eadg3587.
20 Y. Wang, Z. Liu, A. Mazumder, C. G. Gianopoulos, K. Kirschbaum, L. A. Peteanu, R. Jin, *J. Am. Chem. Soc.* **2023**, *145*, 26328–26338.
21 X. Liu, J. Yuan, C. Yao, J. Chen, L. Li, X. Bao, J. Yang, Z. Wu, *J. Phys. Chem. C* **2017**, *121*, 13848–13853.
22 X.-K. Wan, X.-S. Han, Z.-J. Guan, W.-Q. Shi, J.-J. Li, Q.-M. Wang, *Nat. Commun.* **2024**, *15*, 7214.
23 Z. Liu, L. Luo, J. Kong, E. Kahng, M. Zhou, R. Jin, *Nanoscale* **2024**, *16*, 7419–7426.
24 M. Suyama, S. Takano, T. Tsukuda, *J. Phys. Chem. C* **2020**, *124*, 23923–23929.
25 Q. Li, C. J. I. Zeman, G. C. Schatz, X. W. Gu, *ACS Nano* **2021**, *15*, 16095–16105.
26 W.-Q. Shi, L. Zeng, Z.-C. Long, Z.-J. Guan, X.-S. Han, F. Hu, M. Zhou, Q.-M. Wang, *J. Phys. Chem. Lett.* **2025**, *16*, 2204–2211.
27 W. Gu, Y. Zhou, W. Wang, Q. You, W. Fan, Y. Zhao, G. Bian, R. Wang, L. Fang, N. Yan, N. Xia, L. Liao, Z. Wu, *Angew. Chem. Int. Ed.* **2024**, *63*, e202407518.
28 Y. Wang, A. Sardar, Z. Liu, C. G. Gianopoulos, G.He, X. Liu, S. Chen, K. Kirschbaum, A. Mazumder, M. Cotlet, D.-e. Jiang, R. Jin, *J. Am. Chem. Soc.* **2025**, *147*, 42752–42757
